# Structural Connectivity Differences Reflect Microstructural Heterogeneity of the Human Insular Cortex

**DOI:** 10.1002/hbm.70231

**Published:** 2025-05-21

**Authors:** Julian Quabs, Nora Bittner, Svenja Caspers

**Affiliations:** ^1^ Institute for Anatomy, Medical Faculty and University Hospital, Heinrich Heine University of Düsseldorf Düsseldorf Germany; ^2^ Institute of Neuroscience and Medicine (INM‐1), Research Centre Jülich Jülich Germany

**Keywords:** human insula, microstructure, multimodal brain mapping, structural connectivity, tractography

## Abstract

The insular cortex is renowned for its multitude of functions, intricate structural connectivity patterns, and complex cytoarchitecture, yet a unified multimodal concept remains elusive. Microstructural parcellations provide a promising mediator to integrate connectome data into a combined structural–functional framework. While in the macaque insula, a clear relationship between anatomical connections and cytoarchitecture is well established, such correlation in the human insula remains unclear. By combining diffusion data from two large cohorts, including 914 and 204 subjects, respectively, as well as probabilistic tractography and the microstructural JulichBrain Atlas, we uncover how microstructural diversity reflects structural connectivity patterns in the human insula. Analyzing the connectivity of 16 cytoarchitectonic areas, we identified six clusters, two in the posterior and four in the anterior insula. Posterior clusters exhibited strong connectivity with temporal, occipital, and parietal areas encompassing auditory, visual, and somatosensory systems. Conversely, anterior clusters were specifically linked with (orbito)frontal areas, such as Broca's area or frontal opercular areas. Together, our data demonstrate that structural connectivity differences are reflected by fundamental principles of microstructural organization in the human insula. Additional whole‐brain connectivity analyses reveal that two distinct areas within the anterior (Id6) and posterior (Id3) human insula may serve as integrative hubs, mediating between higher‐order cognitive and limbic systems, as well as across sensory modalities. All clusters are openly available in MNI space to support future multimodal studies addressing the relations between cytoarchitecture, structure, functions, and pathologies in this complex region of the human neocortex.

## Graphical abstract

Combining the microstructural JulichBrain Atlas with tractography, we analyzed connectivity patterns within the cytoarchitecture of the human insula. We discovered six clusters, consistent across two large cohorts, with distinct connectivity patterns for different anatomical and functional target regions, reflecting fundamental principles of microstructural organization within the human insula. Additional whole‐brain connectivity analyses reveal that two distinct areas within the anterior (Id6) and posterior (Id3) human insula may serve as integrative hubs, mediating between higher‐order cognitive and limbic systems, as well as across sensory modalities.

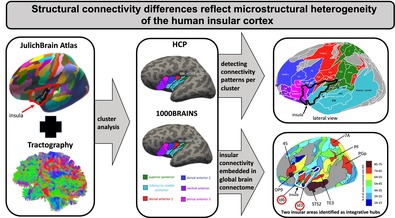

**Summary**

- Connectivity analysis of microstructural areas reveals six distinct clusters within the human insula.
- Connectivity patterns differ both between and within the posterior clusters, which are linked to varying degrees to auditory, visual, and somatosensory systems, and the anterior clusters, which are associated with different higher cognitive and limbic target areas.
- Results are validated across two cohorts.
- Clusters are publicly available in MNI space and can be used for further disentangling the structural–functional relationship in the human insula.
- Two areas of the human insula (Id6 and Id3) may serve as integrative hubs within the whole‐brain structural connectome.

## 1 Introduction

The human insula cortex is a multi‐integrational hub region, intertwining a diverse cytoarchitectonic and structural organization across a variety of functional systems, from pain and interoceptive perception (Craig 2003; Hassanpour et al. 2018; Khalsa et al. 2018) to emotional processing (Zhang et al. 2019; Lotze 2024) and salience detection (Uddin 2015). However, a holistic multimodal conceptualization of this complex brain region is still missing, particularly in the context of its increasing significance as a neurobiological substrate in most psychiatric disorders (Goodkind et al. 2015; Nord et al. 2021; Taylor et al. 2023). In other brain regions such as the parietal lobe (Caspers and Zilles 2018) or parietal operculum (Eickhoff et al. 2010), microstructural parcellations have proven effective as integrating mediators across various modalities (Caspers et al. 2013; Bludau et al. 2018; Amunts et al. 2020). Recent stimulation and imaging studies provide initial evidence that a microstructural framework can also be utilized to integrate functional data of the human insula (Mazzola et al. 2014; Grodzinsky et al. 2020; Duong et al. 2023), while it remains unclear whether and how the underlying structural connectivity could also be integrated in such a framework.

In non‐human primates, the anatomical connectivity and the microstructural heterogeneity of the insula are closely related. Tract tracing studies demonstrated widespread connections between the insula and essential brain regions associated with limbic functions, cognition, motor control, and all sensory modalities, extending across all lobes and most subcortical structures such as the amygdala, hippocampus, claustrum, basal ganglia, and the thalamus (for review, see Mufson et al. (1981), Mufson and Mesulam (1982), Mesulam and Marsel (1982), Mufson and Mesulam (1984), Mesulam and Mufson (1985) and J. R. Augustine (1985), R. Augustine (1996)). A most influential microstructural model of the primate insula has been introduced by Mesulam and Mufson (1985, 1982), dividing it into four sectors based on the cell packing density in layer IV—the concept of the granular shift. While the agranular and granular sectors predominantly connect with regions of similar granularity, the dysgranular sector is linked to areas regardless of their granularity (Mesulam and Mufson 1985). Each sector also presents distinct connectivity with subcortical structures (Carmichael and Price 1995; Chikama et al. 1997; Amaral and Price 1984). Evrard et al. (2014) and Evrard (2019) have recently put forth a finer parceled microstructural map of the macaque insula, further dividing the granularity‐based framework (Mesulam and Mufson 1985) into four granular, four dysgranular, and seven agranular areas, characterized by distinct connectivity patterns and functional properties. Granular areas are proposed to process information from interoceptive, auditory, and vestibular pathways, while agranular areas are characterized by different connectivity profiles, particularly with limbic, visceromotor, and olfactory networks. Dysgranular areas are hypothesized to differ in connectivity, especially with motor and somatosensory systems, as well as subcortical and limbic structures. However, cross‐species comparisons with humans have been challenging, primarily due to methodological disparities, such as the infeasibility of employing tract‐tracing techniques on human subjects.

MRI‐based tractography has provided a systematic approach to explore the anatomical connectivity of the human insula. For instance, Cerliani et al. (2012) reported widespread connections similar to those in macaque brains, with a gradual transition of connectivity patterns from the posterior to anterior direction. Other studies suggest distinct connectivity differences between (i) macroscopically defined segments such as the anterior and posterior insula (Cloutman et al. 2012; Jakab et al. 2012; Denis et al. 2016) and (ii) functionally defined subregions in the posterior, dorsal anterior, and ventral anterior insula (Nomi et al. 2018; Klugah‐Brown et al. 2023). Ghaziri et al. (2017) and Ghaziri et al. (2018) employed a fine‐grained subdivision into 19 parcels to unveil topographically dependent connectivity disparities of the insula across cortical and subcortical targets. The region‐specific fiber tract architecture within the insula also shows significant alterations in conditions such as epilepsy (Obaid et al. 2021), stroke (Klepzig et al. 2023), and depression (Fu et al. 2021), emphasizing the clinical importance of insula‐related fiber bundles as possible mediators of functional deficits and the need for connectivity analysis of biologically pertinent subunits within this brain region.

In parallel to tractography studies, recent developments in microstructural brain mapping reported that the human insula can be divided into 16 distinct areas (Kurth, Eickhoff, et al. 2010; Grodzinsky et al. 2020; Quabs et al. 2022; Hein, n.d.). Similar to the macaque brain, the granular shift represents an important cytoarchitectonic organizational principle of the human insula. However, both the macaque insula (Evrard et al. 2014; Evrard 2019) and the human insula also show a remarkable microstructural diversity beyond the properties of Layer IV (Quabs et al. 2022). Implemented within the JulichBrain Atlas (Amunts et al. 2020), these new maps serve as an anatomical framework in standard reference space, allowing, for example, the use of microstructural areas as seed regions in structural connectivity analysis.

Among the few studies that have explored tractography of the human insula, only Cerliani et al. (2012) investigated the connectivity between the insula and microstructural target areas, proposing a potential link between cytoarchitecture, connectivity, and functional organization similar to the macaque brain. Yet, connectivity patterns of distinct cytoarchitectonic areas of the human insula itself have not been investigated in detail. The present study was designed to address this important unknown by combining tractography with microstructurally defined areas of the human insula across two large cohorts, one serving as a discovery and one as a replication sample. We here seek to unravel the functionally relevant network integration of different insular areas by means of their structural connectivity, further disentangling the potential role of microstructure as an integrating mediator across different modalities and pathologies of the human insular cortex. We further aim at clarifying the insula's role as an integrative hub region by assessing insular microstructural connectome data within a framework of whole‐brain cytoarchitectonic connectivity.

## 2 Materials and Methods

**FIGURE 1:**
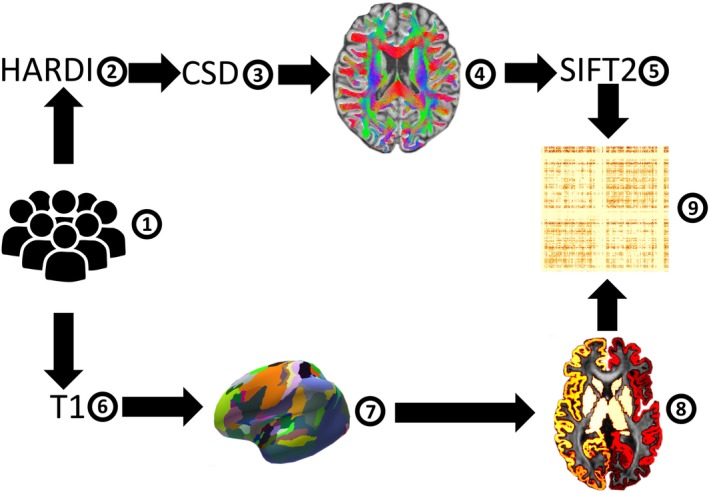
Calculating connectivity strength of microstructural areas of the human insula. High angular resolution diffusion images were used from 914 subjects from the population‐based 1000BRAINS cohort and 204 subjects from the Human Connectome Project (1, 2). Constrained spherical deconvolution was employed as local diffusion model using MRtrix3.0 (3). White matter fiber paths were reconstructed through probabilistic, anatomically constrained streamline tractography (4). Each streamline was assigned a weight to align the total streamlines' density with the diffusion model fiber density estimate for each fixel (5). Next, the microstructural Julich Brain Atlas was overlaid onto the T1 image of each subject, generating a mask for connectome matrix reconstruction (6–8). The connectivity strength between insular areas and all other regions of the Julich Brain Atlas was computed as fiber bundle capacity (FBC) in a connectivity matrix (9).

In this study, we employed a standard tractography framework across two large cohorts, as illustrated in Figure 1 and in detail explained in the subsequent sections.

### 2.1 Subject Data

#### 2.1.1 1000BRAINS Study

The participants in the subsequent analyses were taken from the 1000BRAINS study (Caspers et al. 2014), designed to investigate the structural and functional variability in the aging brain. Recruitment was sourced from the Heinz Nixdorf Recall (HNR) Study (Schmermund et al. 2002; Erbel et al. 2012) and the HNR MultiGenerationStudy, utilizing a population‐based sample randomly selected from German citizen registries in Essen, Bochum, and Mülheim. To obtain age‐specific characteristics at the general generation level, all participants who were eligible for MRI measurements were included. Of the available 1315 subjects, 401 were excluded from the present study because of missing diffusion data. A final sample of 914 participants (431 female, 483 male, *M*_age_ = 59.9 years, SD_age_ = 13.66) was used for further analyses, whereby this sample served as the discovery sample for the current study. The study protocol of 1000BRAINS was approved by the Ethics Committee of the University of Essen, Germany, and all subjects provided written consent prior to inclusion.

#### 2.1.2 Human Connectome Project Data Set

All results were cross‐validated and replicated using a second independent data set, that is, analyzing 204 preprocessed adult subjects (102 female, 102 male, between 22 and 35 years) from the Human Connectome Project (Van Essen et al. 2013)—release version s900.

### 2.2 Image Acquisition and Processing

#### 2.2.1 Imaging 1000 BRAINS Study

Magnetic resonance imaging was performed using a 3 T Siemens TimTRIO MR scanner with a 32‐channel head coil (Erlangen, Germany). Different sequence images were included in the current study for investigating the structural connectivity (see Caspers et al. (2014) for a detailed description of the 1000BRAINS study protocol): (i) For surface reconstruction, a three‐dimensional high‐resolution T1‐weighted magnetization‐prepared rapid acquisition gradient‐echo (MPRAGE) anatomical scan was acquired (176 slices, slice thickness 1 mm, repetition time [TR] = 2250 ms, echo time [TE] = 3.03 ms, field of view [FoV] = 256,256 mm^2^, flip angle = 9°, voxel resolution 1 × 1 × 1 mm^3^). (ii) For structural connectivity analyses, high‐angular resolution diffusion imaging (HARDI) data with the following parameters were used: (1) 120 directions data set; EPI, TR = 8 s, TE = 112 ms, 13 b0‐images (interleaved), 120 images with *b* = 2700 s/mm^2^, voxel resolution = 2.4 × 2.4 × 2.4 mm^3^; (2) 60 direction subset (out of 120 direction data set); EPI, TR = 6.3 s, TE = 81 ms, 7 b0‐images (interleaved), 60 images with *b* = 1000 s/mm^2^, voxel resolution = 2.4 × 2.4 × 2.4 mm^3^.

#### 2.2.2 Imaging Human Connectome Project Data Set

Image data were acquired on a customized Siemens Magnetom Skyra 3 T MRI system. For the validation, we used corresponding image sequences as for the 1000BRAINS Study: (i) high resolution T1 anatomical images were obtained using the 3D magnetization‐prepared rapid gradient echo sequence (MPRAGE) (Mugler and Brookeman 1990) (with 0.7 × 0.7 × 0.7 mm^3^ voxel size, TR/TE = 2400/2.14 ms, and flip angle = 8°). (ii) the diffusion imaging protocol included three diffusion‐weighted shells (*b* = 1000, 2000, and 3000 s/mm^2^), 90 diffusion‐weighted volumes each, 18 reference volumes (*b* = 0 s/mm^2^), and reversed phase encoding for distortion correction (Andersson et al. 2003) with the following imaging parameters: 145 × 145 matrix, 174 slices, 1.25 × 1.25 × 1.25 mm^3^ voxel size, TR/TE = 5520/89.5 ms. Surface reconstruction and streamline tractography followed the same procedures as used for the 1000BRAINS data set and were consistent with previously conducted tractography studies on the HCP cohort (Civier et al. 2019).

#### 2.2.3 Surface Reconstruction

3D images were processed using the automated surface‐based pipeline of the FreeSurfer Software package (version 6, Athinoula A. Martinos Center for Biomedical Imaging). Comprehensive procedural details were outlined by Dale et al. (1999) and Fischl (2012), along with documentation available at http://surfer.nmr.mgh.harvard.edu. The processing steps encompassed motion correction, intensity normalization, removal of extra‐cerebral voxels (nonbrain tissue), spatial normalization, volumetric segmentation, and cortical surface reconstruction. Cortical surface reconstruction involved generating the white surface at the boundary of white matter and gray matter, followed by the creation of the pial surface at the gray matter‐cerebrospinal fluid interface. The resulting mesh model of the pial surface was triangulated, comprising approximately 120,000 vertices per hemisphere with an average surface area of 0.5 mm^2^.

#### 2.2.4 Streamline Tractography

For this study, we employed streamline tractography in accordance with standard pipelines, as used, for example, in the Human Connectome Project (www.humanconnectomeproject.org) or the UK Biobank (www.ukbiobank.ac.uk). Initially, for each participant, tissue probability maps (TPMs) delineating grey matter (GM), white matter (WM), and cerebrospinal fluid (CSF) were generated from T1‐weighted data using the Computational Anatomy Toolbox (Gaser et al. 2022) within SPM12 (Gaser et al. 2022). Next, diffusion‐weighted imaging (DWI) data were corrected for eddy current and motion artifacts, including interpolation of slices affected by signal dropouts (Andersson et al. 2016; Andersson and Sotiropoulos 2015). DWI data were then rigidly aligned to T1 space, followed by the computation of Anisotropic Power Maps used for nonlinear transformation of TPMs into the diffusion space (Dell'Acqua et al. 2013). All transformation processes were conducted using the Advanced Normalization Tools (ANTs) version 2.1.1 (Avants et al. 2014). Finally, the data sets with *b*‐values of 1000 and 2700 were merged into a unified file and corrected for variations in echo times.

Following the preprocessing procedure, local modeling and probabilistic anatomically constrained streamline tractography were performed using the MRtrix software package version 0.3.15 (Tournier et al. 2012; Smith et al. 2012). The constrained spherical deconvolution (CSD) local model was computed utilizing multi‐tissue CSD of multi‐shell data across all shells, with a maximal spherical harmonic order of 8. Subsequently, 10 million streamlines were generated with dynamic seeding at the grey–white matter interface for each participant, employing the probabilistic iFOD2 algorithm with a maximal length of 250 mm and a cutoff value of 0.06.

### 2.3 Connectivity Analysis

#### 2.3.1 Connectome Matrices

For the construction of the connectome matrices, we used the areas defined in the microstructural JulichBrain Atlas (Amunts et al. 2020) as input nodes. First, the surface‐based atlas was projected from the fsaverage template onto the T1 image of each subject and converted into a volume. This ensured anatomical accuracy, as the volume of each area remained within the pial surface and white matter boundary, without extending into the white matter or subcortical structures (Figure 1). The resulting volumes of areas were then used as nodes for connectome reconstruction. Next, the mask was rigidly transformed into the diffusion space employing fsl (Jenkinson et al. 2012). To increase the biological accuracy of the tractogram, each streamline was assigned a weight aligning the total streamlines density traversing each fixel (Dhollander et al. 2021) and the actual fiber volumes estimated from the diffusion model (Smith 2020). The resulting fiber bundle capacity (FBC) (Smith 2020) is defined as the sum of intra‐axonal cross‐sectional areas of these fibers reflecting the capacity to carry information between two regions of interest (ROIs). The JulichBrain Atlas in diffusion native space, the whole‐brain tractogram, and the SIFT2 weights per streamline were then fed into tck2connectome (MRtrix 0.3.15). This resulted in a symmetric 214 × 214 matrix which contained the volume‐corrected FBC per ROI combination per subject.

#### 2.3.2 Statistical Analysis

For statistical analysis, ROI‐specific connectivity matrices of all microstructural insular areas were extracted from the connectome matrix and combined for both hemispheres, because of the high number of ROIs and seeds used in this study. Connectivity strength between each combination of an insular area and a microstructural target area was represented by the trimmed mean FBC, to eliminate the effect of outliers and estimate an average across all subjects.

To test connectivity differences between areas, we compared the true mean FBC for each area for a specific target region to a null distribution of means. The distribution was generated by random sampling and averaging FBC values from the total subject's pool for this target region across all areas. The procedure was iterated 10,000 times. We accepted the result as significant if the true mean exceeded 99% of the random distribution (*p* = 0.01). To examine if the connectivity strength is also significantly increased compared to the entire insula connectome, we sampled another null distribution of means, deriving from the total subject's pool for all target regions and areas (*p* = 0.01).

Next, the microstructural areas were further examined with regard to connectivity differences and similarities by means of a hierarchical cluster analysis, k‐means clustering, and multidimensional scaling (MDS) analysis. We included target areas as relevant features only if at least one insular area exhibited significantly increased connectivity (Bluma and Langley 1997). Given the high dimensionality of our data, we used the Manhattan distance (Aggarwal et al. 2001) with the average linkage method (Jarman 2020) to quantify connectivity differences between areas and the cophenetic correlation as a goodness‐of‐fit measure for the resulting cluster solution (Gere 2023). For the *k*‐means clustering, we used the Elbows method (Umargono et al. 2019) to determine the optimal number of clusters and projected the results onto the MDS plot. We repeated the same procedure with the identified clusters to explore similarities and differences between the anterior and posterior insula. Connectivity profiles of detected groups were visualized (i) by depicting the mean FBC between clusters and targets in a polar plot, (ii) by projecting the color‐coded mean FBC for the cluster with the highest connectivity strength per target area on the fsaverage template, and (iii) by computing group‐averaged tract density maps in MNI 152 standard space. For further anatomical interpretation within an established white matter framework, tract density maps were also compared with the Johns Hopkins University and deep/superficial white matter atlases (Wakana et al. 2007; Guevara et al. 2012, 2017). All detected clusters are publicly available in MNI reference space (see Data Availability Statement).

Subsequently, we tested the most pertinent anatomical and functional target areas against the null hypothesis, that the clusters do not differ in connectivity patterns, which would correspond to lacking evidence for a biologically meaningful interpretation of our cluster solution. Based on the visualization of cluster connectivity and bootstrapping, as described above, we initially identified the most strongly connected anatomical target areas across clusters. Additionally, microstructural target areas were merged into a set of functionally relevant target regions (Table 1). For all target regions, a one‐sided *t*‐test was conducted to compare the cluster with the highest connectivity strength for a specific target against all other clusters. Given the limitations of *p* values in large data sets, we only rejected the null hypothesis if the tested cluster significantly outperformed all others (*p* = 0.01) with an effect size > 0.8 (Lin et al. 2013).

**TABLE 1:** Grouping of target areas in relation to anatomical and functional criteria. For the topographical localization of the defined regions (see Figure 7A).

Group name	Included areas
Frontal	Frontal_to_Temporal_I, Frontal_to_Temporal_II, Frontal_I, Frontal_II IFS1, IFS2, IF3, IFS4, IFJ1, IFJ2, 8v2, 8v1, Fp1, Fp2, OP5, OP6, OP7, OP8, OP9, 8d2, 8d1
Broca	44, 45
Orbitofrontal	Fo1, Fo2, Fo3, Fo4, Fo5, Fo6, Fo7
Motor	6d1, 6d2, 6ma, 6d3, 6mp, 4p, 4a
Auditory	TE1.0, TE1.1, TE1.2
Primary visual	hOc1, hOc2
Higher visual	hOc3v, hOc3d, hOc4v, hOc4d, hOc4lp, hOc4Ia, hOc5, hOc6
Primary somatosensory	1, 2, 3a, 3b
Secondary somatosensory	OP1, OP2, OP3, OP4

To validate our findings, we first applied hierarchical clustering, *k*‐means clustering, and MDS to the HCP data set. The statistical analysis was replicated with the resulting cluster solution, including the calculation of trimmed mean FBC, bootstrapping, and statistical testing of the most significant anatomical and functional target regions. Connectivity patterns were directly compared between the HCP data set and the 1000BRAINS cohort and depicted in a polar plot. We considered the connectivity profiles similar for a target area across data sets if both exceeded the bootstrapping threshold for this specific target region. The most significant target regions were separately tested for cluster‐specific connectivity and compared for both cohorts. All statistical analyses were performed using R (version 4.2.1).

### 2.4 Comparison of the Insula Connectome With Whole‐Brain Connectivity

To investigate how the structural connectome of the insula compares to that of the rest of the brain, we compared microstructural connectivity profiles across the entire JulichBrain Atlas (Version 2.9). Structural connectivity for each microstructural area was quantified as the trimmed mean FBC between the ROI and all other atlas parcels. These measures were derived from the 1000BRAINS cohort, following the methodology described above. To preserve and visualize the global topological structure of the high‐dimensional connectivity data, including similarities and differences between profiles, we applied Uniform Manifold Approximation and Projection (UMAP) (McInnes et al. 2020) to generate a two‐dimensional representation of the underlying connectivity manifold. Additionally, we computed weighted edges between data points to reflect how similar points are based on proximity in the original feature space.

Next, we aimed to directly assess the underlying structural connectome of the microstructural areas, with a particular focus on their integrative properties. This analysis was motivated by the hypothesis that highly integrative areas—such as those proposed within the insular cortex—may not exhibit a few strong connections but instead maintain weaker connections with a wide range of brain areas. We operationally defined “integrative connectivity” as a connectivity profile characterized by broadly distributed, “limited‐strength” connections across multiple areas. Specifically, a connection between two areas was considered to be of “limited strength” if it ranked within the top 50% of the target area's connectivity profile, excluding the strongest connections as determined by a bootstrapped threshold for each target area. Results were projected onto the cortical surface and visualized using the NIH color scheme, ranging from blue to red. For example, a light red ROI indicates that the respective ROI showed limited‐strength connections with approximately 65%–74% of all other areas in the Julich‐Brain Atlas. ROIs coded in red therefore exhibited limited‐strength connectivity with a broad array of targets, suggesting a potentially integrative role for these areas. To additionally visualize connectivity differences between insula‐associated integrative areas, “integrative connectivity” profiles of insular areas were projected onto a cortical surface. Each parcel was categorized as connected to: (i) a single insular integrative area (if only one was in the top 50% of its target regions), (ii) all insular integrative areas (if all were in the top 50%), or (iii) none (if none was in the highest 50%).

The combination of UMAP embeddings and integrative connectivity maps provided a comprehensive framework for situating the structural connectome of the insula within the broader context of whole‐brain connectivity. All analyses were performed using Python (v3.12.8), with the UMAP package for dimensionality reduction and the nilearn package for surface projections.

## 3 Results

### 3.1 Connectivity Differences Between Microstructural Areas of the Human Insula

#### 3.1.1 Connectivity Patterns of Microstructural Insular Areas

**FIGURE 2:**
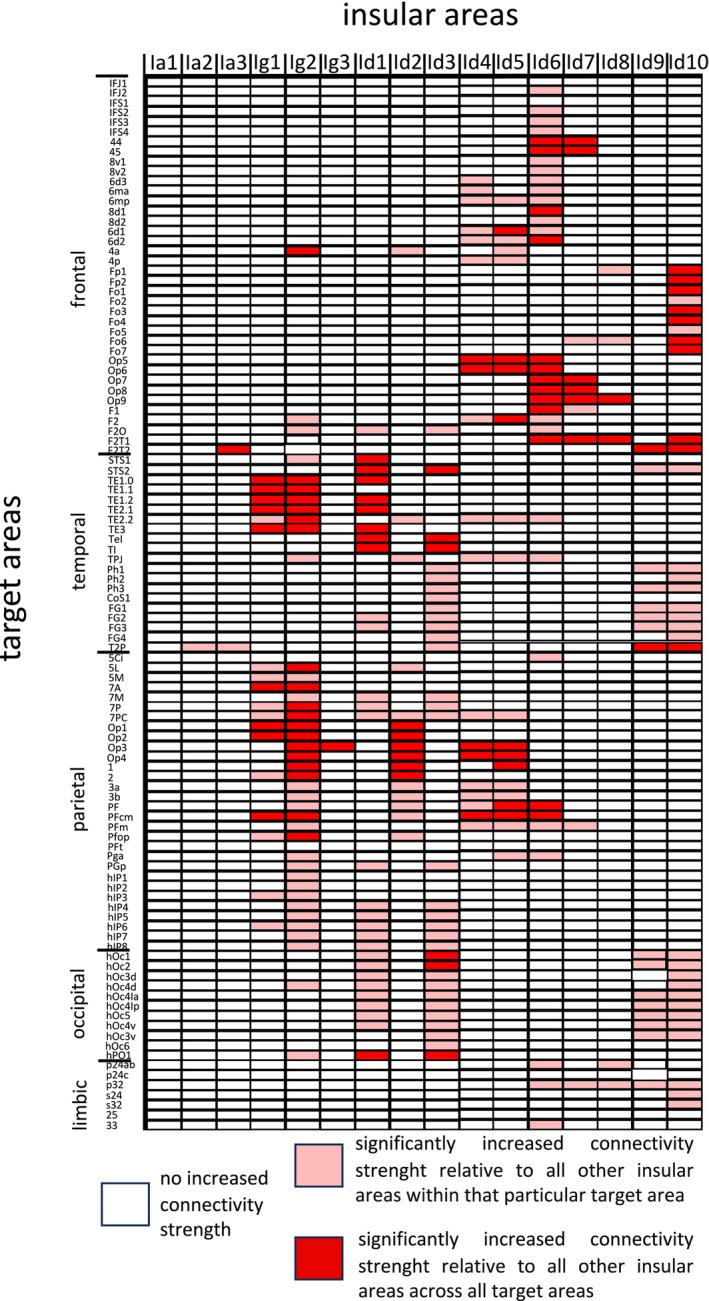
Connectivity map illustrating connectivity strength between insular areas and all other areas of the microstructural Julich Brain atlas. A light red color denotes that the corresponding connectivity value exceeds the target area‐specific threshold, thereby indicating significantly increased connectivity strength for that insular area relative to all other insular areas within that particular target area. A dark red color indicates that the connectivity value additionally exceeds the threshold for a large effect size, showing that the connectivity strength is significantly increased not only compared to all other insular areas for this specific target but also compared to all other target areas (see Section 2 for details).

To investigate whether the microstructural organization of the human insula reflects the underlying structural connectivity, we combined the cytoarchitectonic JulichBrain Atlas with tractography. The connectivity strength of the areas for each target region was tested for area‐specific connections by comparing it to a bootstrapped null distribution (Figure 2, Table S1).

Agranular areas (Ia1, Ia2, and Ia3) and granular area Ig3 in the posterior insula showed no specific connectivity with any target area beyond the connectivity observed for insular areas in general, while all other insula areas exhibited distinct connectivity patterns. Granular posterior areas Ig1 and Ig2 were characterized by increased connectivity with the planum temporale, especially auditory target regions (Te1.0, Te1.1, Te1.2, Te2.1, and Te2.2), and microstructural areas localized in the anterior parietal lobe such as somatosensory cortex areas 1 and 2, as well as parietal opercular areas Op1, Op2, Op3, and Op4. Dysgranular posterior areas Id1 and Id3 both showed enlarged connectivity with visual‐related areas in the occipital lobe and adjacent parietal areas, whereas insular area Id1 additionally showed higher FBC with areas of the inferior temporal gyrus and Id3 with areas of the superior temporal gyrus. Connectivity patterns of the insular area Id2 comprised increased FBC with parietal opercular and primary somatosensory areas. While areas of the posterior insula were primarily linked to the parietal, temporal, and occipital lobes, anterior insular areas exhibited strong connections with the frontal lobe. Area Id6 showed the most widespread connections across the entire frontal lobe, except for the orbitofrontal cortex, while area Id7 expressed heightened connectivity with Broca's area 44 and 45 as well as frontal opercular areas. Areas Id8 and Id9 expressed only a few unspecific connectivity patterns with frontal and parietal areas, whereas area Id10 exhibited an exclusively strong connectivity with the orbitofrontal cortex. Notably, Id4 and Id5 were the only areas demonstrating increased structural connectivity with both the frontal and parietal operculum, thereby exhibiting connectivity properties of both anterior and posterior insular areas.

These results indicate the presence of distinguishable connectivity patterns among groups of microstructural areas, without a singular connectivity pattern unique to each specific area. One notable exception is area Id10, which was the only area that exhibited an exclusively strong connection with the orbitofrontal cortex.

#### 3.1.2 Cluster Solution

**FIGURE 3:**
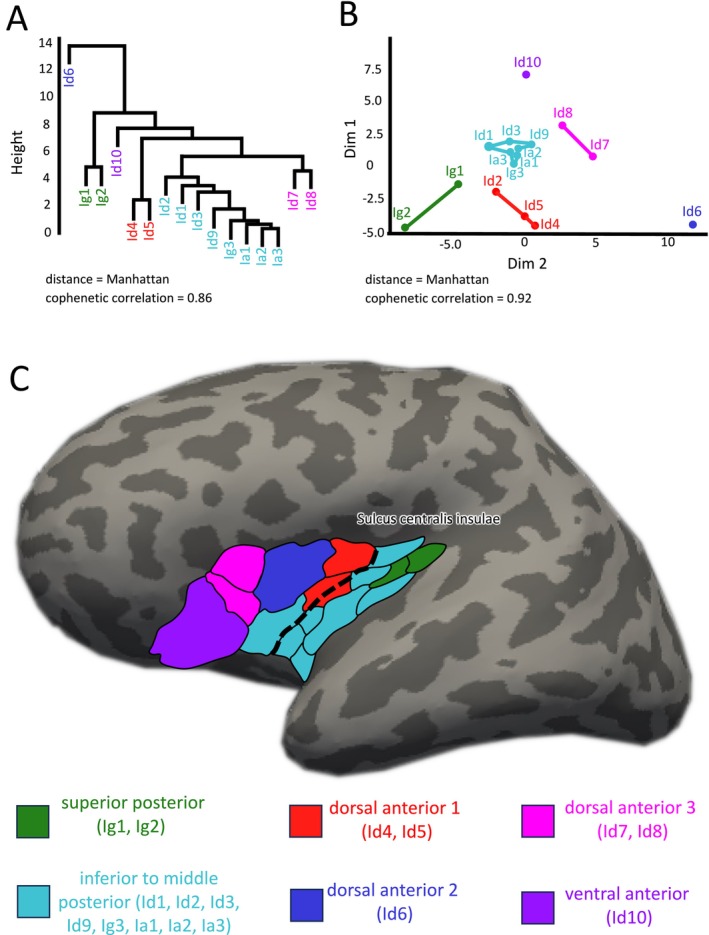
Clustering connectivity patterns of insular microstructural areas utilizing the 1000BRAINS cohort. Both hierarchical cluster analysis (A) and k‐means clustering based on multidimensional scaling (B) reveal nearly identical cluster solutions in six different groups. Clusters are named based on their topographical localization in the insula, illustrated in (C).

**FIGURE 4:**
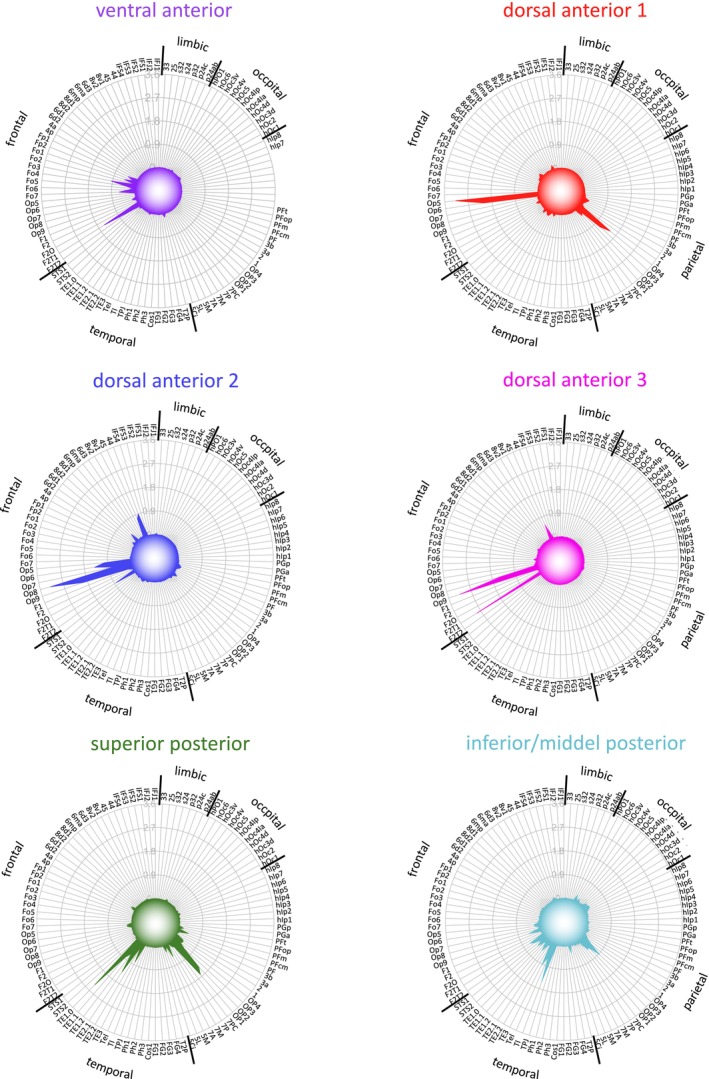
Connectivity strength between insular clusters and all other areas of the microstructural Julich Brain atlas. The polar plot captures the connectivity fingerprints of each cluster and the differences between them.

**FIGURE 5:**
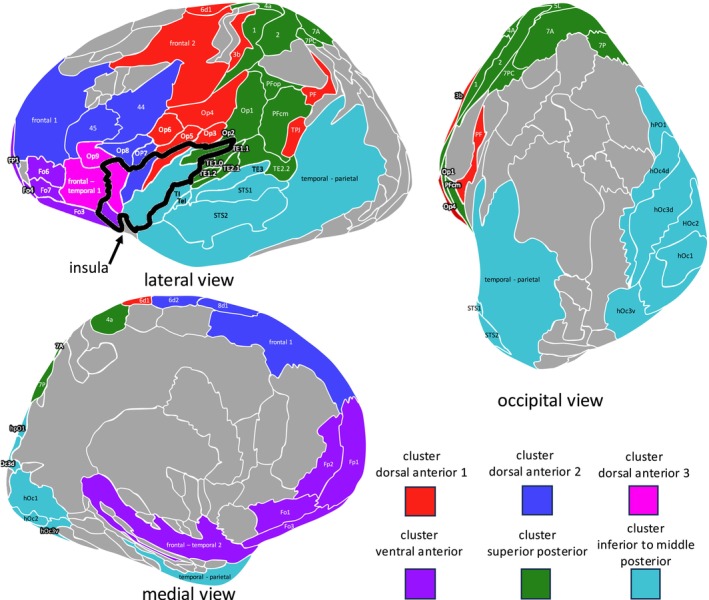
Visualization of connectivity fingerprints for each cluster projected onto the fsaverage template. The color‐coded representation of areas signifies the predominant cluster with the highest fiber bundle capacity in the respective area. The intensity of the coloring reflects the actual strength of connectivity.

Next, we further examined the microstructural areas with regard to connectivity differences and similarities by means of hierarchical cluster analysis and MDS. Both approaches indicated an optimal cluster solution in six groups with high goodness of fit indicators (Figures 3 and S1). Three clusters were located in the dorsal anterior insula, one cluster in the ventral anterior insula, and two clusters in the posterior insula (Figure 3C). Each cluster presented a unique connectivity pattern (Figures 4 and 5).

Dorsal anterior cluster 1 demonstrates the strongest connectivity with frontal and parietal opercular areas (OP3, OP4, OP5, and OP6) (Figures 4 and 5), while dorsal anterior clusters 2 and 3 exhibited widespread connections across the frontal lobe such as Broca's areas and frontal opercular areas, varying in connectivity strength. The ventral anterior cluster exhibits exclusive connectivity with orbitofrontal areas. In the posterior insula, the superior posterior cluster displays strong connections with the parietal operculum (Op1 and Op2) and primary auditory areas, while the inferior to middle cluster is linked with visual‐related regions and microstructural areas in the middle and superior temporal gyrus. Upon clustering all groups collectively, a cluster number of *k* = 4 appeared as the optimal solution (Figures S2 and S3). It became evident that posterior insular clusters exhibited a higher degree of similarity compared to the anterior clusters, and therefore, the anterior insula expressed more heterogeneous connectivity patterns. Especially, dorsal anterior cluster 1 seemed to be more dissimilar compared to other anterior clusters, likely due to its bridging connectivity between frontal and parietal lobes. For an overview of FBC between clusters and all target areas (see Table S2).

The underlying fiber tracts, reconstructed as tract density maps (Figure S4), closely adhered to the expected white matter architecture within their respective anatomical locations. Fibers from all clusters were observed to originate or terminate in the extreme capsule. The majority of high‐probability voxels in the tract density maps were attributed to short association fibers.

Dorsal anterior cluster 1 was primarily associated with superficial white matter tracts connecting the insula and operculum to the precentral and postcentral gyri, as well as the supramarginal gyrus. Fibers within dorsal anterior clusters 2 and 3 traversed the genu of the corpus callosum and also extended toward the temporal and occipital cortices via long‐range association pathways, including the arcuate fasciculus and the inferior longitudinal fasciculus. The voxels with the highest tract probability, however, were localized to short‐range association fibers within the frontal lobe, connecting the insula with the inferior frontal gyrus, the operculum, and the superior to middle frontal gyri. The ventral anterior cluster was primarily associated with short association fibers linking the insula and the orbitofrontal cortex, as well as fibers between the orbitofrontal cortex and the middle frontal gyrus. Additionally, fibers were part of long‐range association tracts such as the uncinate fasciculus and the inferior fronto‐occipital fasciculus. The superior posterior cluster was assigned to the splenial portion of the corpus callosum and encompassed fibers connecting the insula to the supramarginal gyrus, as well as fibers linking the precentral and postcentral gyri and the transverse temporal gyrus to the superior temporal gyrus. Finally, the inferior‐to‐middle posterior cluster followed the course of the inferior longitudinal fasciculus into the deep occipital white matter. This cluster also included fibers of the uncinate fasciculus, connections between the insula and the supramarginal gyrus, and short association fibers interconnecting the superior, middle, and transverse temporal gyri.

Overall, these findings support the hypothesis that differentiable connectivity patterns in the human insula are organized across groups of microstructural areas rather than being unique to each cytoarchitectonic area.

#### 3.1.3 Connectivity Differences Between Clusters

**FIGURE 6:**
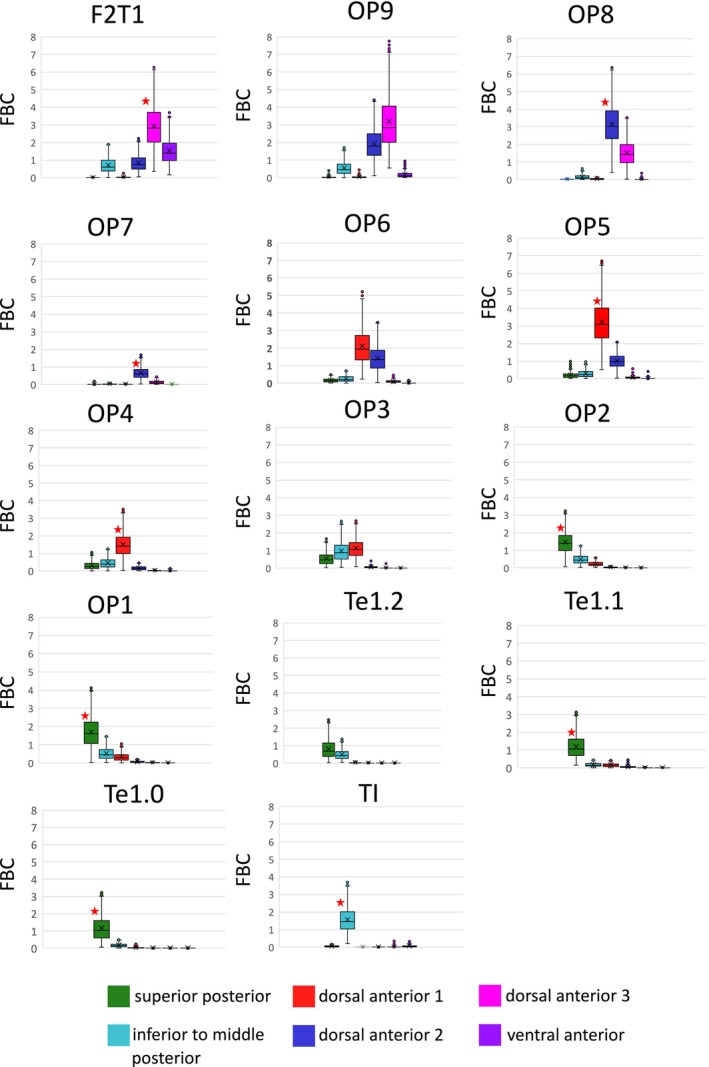
Connectivity strengths between insular clusters and opercular target areas. The connectivity strength for each cluster and opercular target area is represented as fiber bundle capacity (FBC) and depicted in a boxplot (outliers excluded). FBC values for each individual subject are provided in Table S5. The cluster exhibiting the highest FBC for each opercular area was statistically tested against the null hypothesis of no difference from the FBC of all other clusters. A red star denotes instances where the FBC of the respective cluster significantly exceeded that of all other clusters, with an effect size greater than 0.8 (see Table S3 for detailed statistics).

The strongest connectivity across the entire insula connectome was observed with microstructural areas of the adjacent parietal and frontal opercula as well as the planum temporale. Among the 14 opercular/planum temporal areas, 9 exhibited unique connections to one of the microstructurally defined clusters (Figure 6, Table S3). Dorsal anterior cluster 2 displayed exclusive connections with frontal operculum areas OP8 and OP7, while dorsal anterior cluster 1 was linked with areas OP5 and OP4. For the posterior insula, the superior posterior cluster demonstrated specific connections with areas OP2, OP1, Te1.0, and Te1.1, whereas the inferior to middle posterior cluster showed exclusive connections with planum temporale area TI.

**FIGURE 7:**
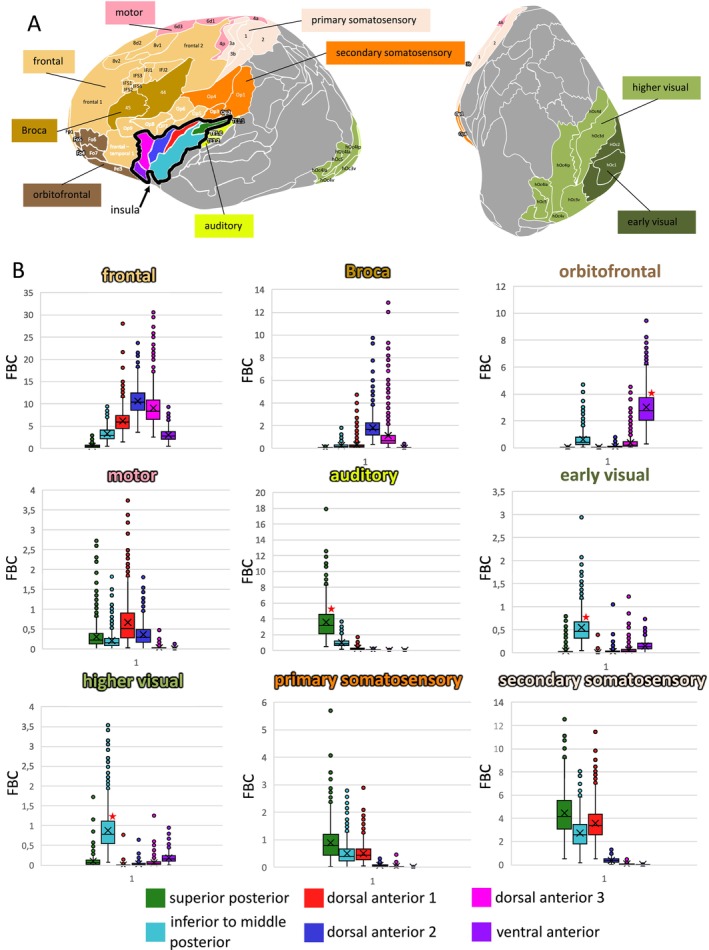
Connectivity strengths between insular clusters and anatomically and functionally relevant target regions. Grouping of target regions is indicated in Table 1 and visualized in (A). The connectivity strength for each cluster and anatomical/functional target region is represented as fiber bundle capacity (FBC) and depicted in a boxplot. FBC values for each individual subject are provided in Table S5. The cluster exhibiting the highest FBC for each anatomical/functional target region was statistically tested against the null hypothesis of no difference from the FBC of all other clusters. A red star denotes instances where the FBC of the respective cluster significantly exceeded that of all other clusters, with an effect size greater than 0.8 (see Table S4 for detailed statistics, Figure S3).

For the functionally relevant target regions, three systems expressed unique connectivity patterns (Figure 7, Table S4): (i) the orbitofrontal cortex with the ventral anterior cluster, (ii) the visual system with the inferior to middle posterior cluster, and (iii) the auditory system with the superior posterior cluster. Although not significant for a specific cluster, somatosensory target regions were exclusively connected with posterior clusters and dorsal anterior cluster 1, while functions associated with the frontal lobe primarily expressed connections with anterior insular clusters. For the average FBC per subject computed for each combination of insular connectivity cluster and functional/anatomical target region for the 1000BRAINS data set (see Table S5).

These data demonstrate that there are distinct connectivity differences between the microstructurally based clusters, further supporting the hypothesis that the cytoarchitectonic organization of the human insula is reflected by the underlying structural connectivity.

### 3.2 Validation of Results in Human Connectome Project Data Set

**FIGURE 8:**
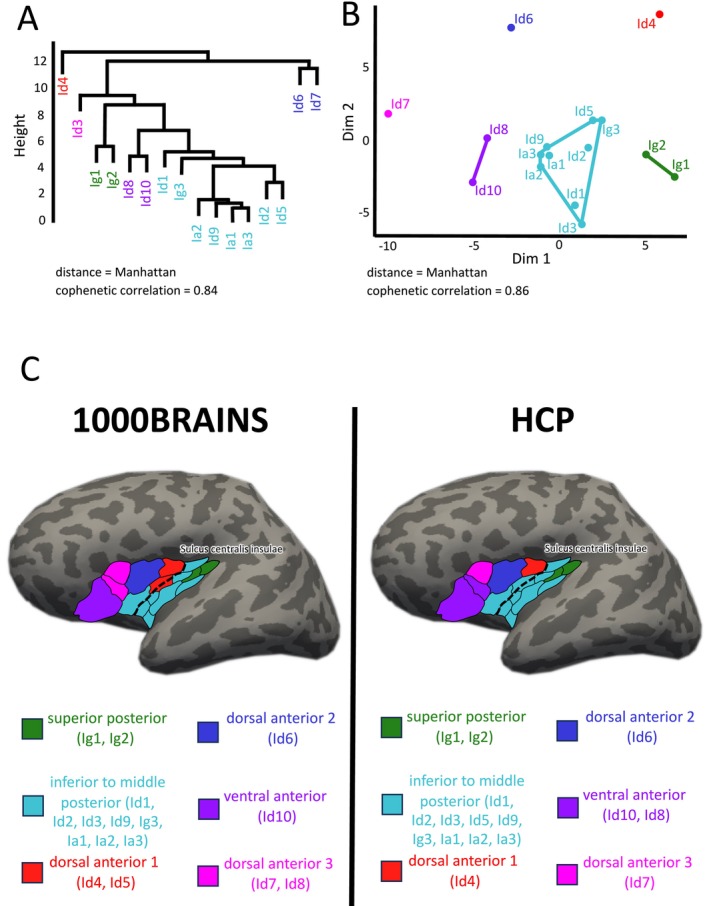
Clustering connectivity patterns of insular microstructural areas utilizing the HCP data set. Both hierarchical cluster analysis (A) and k‐means clustering based on multidimensional scaling (B) reveal nearly identical cluster solutions in six different groups (Figure S1). The topographical localization of clusters as identified by the MDS was compared to the findings from the 1000BRAINS cohort, illustrated in (C).

To cross‐validate our findings, we applied the same procedure on 204 subjects from the HCP data set. Cluster analysis of insular areas identified an optimal solution at *k* = 6 (Figure S1), with clusters showing similar anatomical localization between the HCP and 1000BRAINS cohort (Figure 8, Table S2). The cluster composition differed in three cases: (i) In the 1000BRAINS cohort, area Id8 clustered with Id7, whereas in the HCP cohort, it clustered with area Id10. Connectivity analysis of individual areas (Table S1) indicated that Id8 and Id10 in the HCP cohort shared a more similar connectivity profile, particularly in orbitofrontal regions. (ii) Hierarchical clustering highlighted a distinct role for area Id3 in the HCP data set, expressing unique connectivity with fusiform gyrus areas (FG1–FG4) that was not visible for the 1000BRAINS cohort. (iii) For the HCP data set, area Id5 showed more similar connectivity with inferior‐to‐middle posterior insular areas, particularly regarding temporal areas, resulting in its clustering with the inferior‐to‐middle posterior group in the HCP data set, rather than with Id4 as in the 1000BRAINS cohort.

**FIGURE 9:**
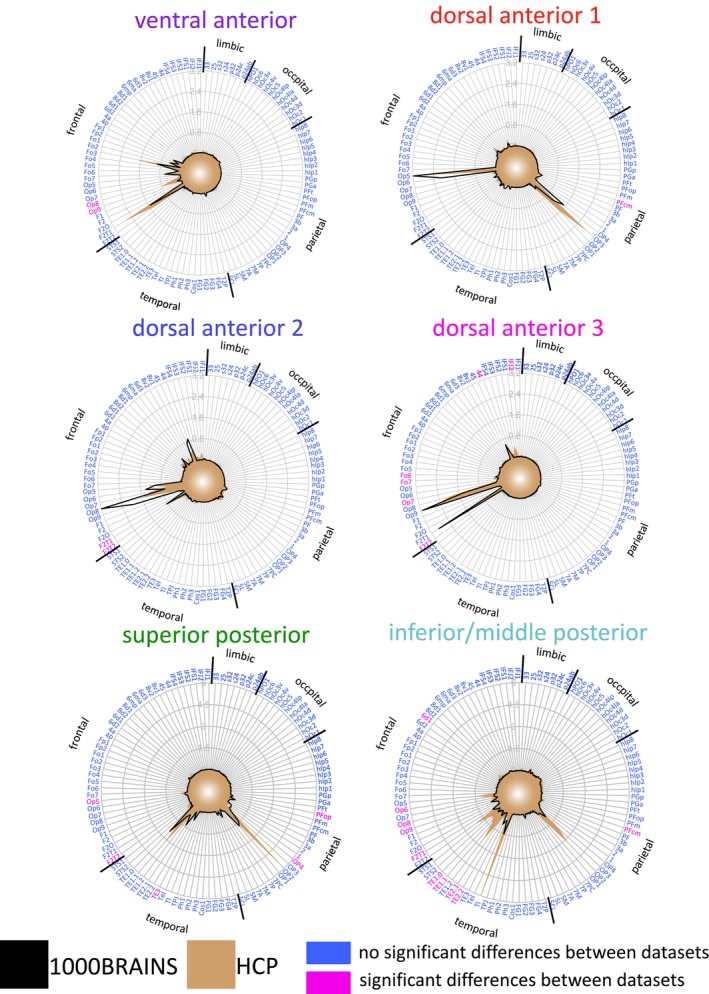
Validation of the connectivity fingerprints for the identified clusters in the 1000BRAINS cohort compared to the HCP data set. A blue coloration of the target area indicates that the cluster has (i) surpassed the target‐specific threshold in both cohorts, thus observing a significantly increased fiber bundle capacity for the respective target area in both data set, (ii) not surpassed the target‐specific threshold in both cohorts, thus observing no significantly increased fiber bundle capacity for the respective target area in both data sets. A magenta coloration of the target area indicates that the cluster surpassed the target‐specific threshold only in one cohort.

**FIGURE 10:**
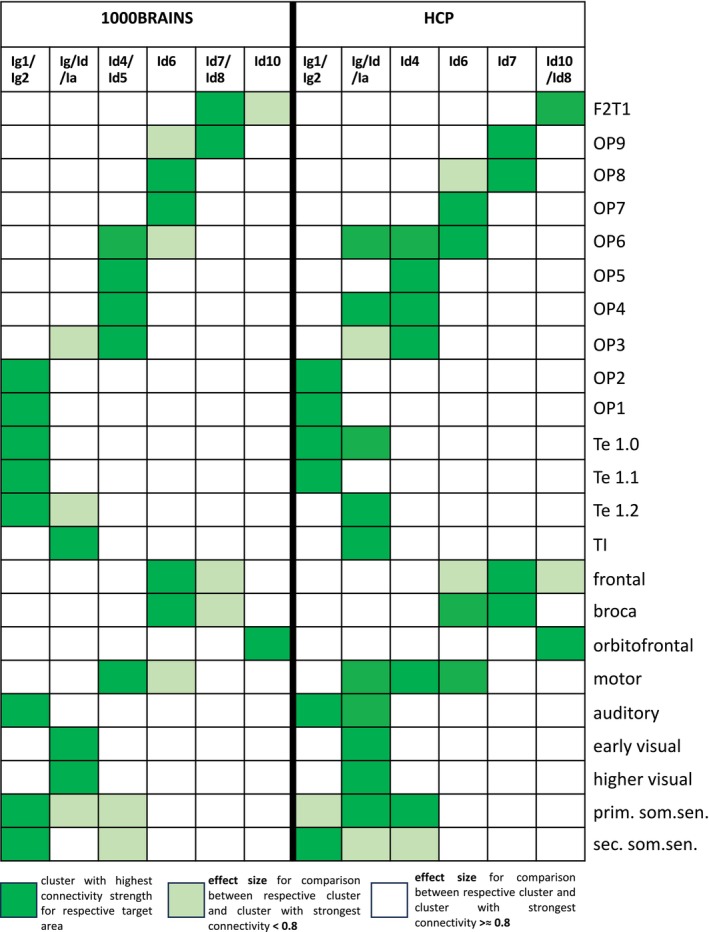
Comparing connectivity patterns of the detected clusters for most relevant anatomical and functional target regions between the 1000BRAINS and HCP data sets. The cluster exhibiting the highest connectivity strength for the respective target (shown in dark green) was tested against all other clusters. The strength of significant results was assessed using the effect size (*d*). A white cell color indicates a large effect size (*d* > 0.8), while light green signifies a small to medium effect size (*d* < 0.8). If two clusters are depicted in dark green for one target area, there is no statistical difference between them. For statistical details, refer to Table S3 (opercular target areas) and Table S4 (functional and anatomical regions).

Comparison of clusterwise connectivity across cohorts revealed nearly identical patterns among clusters, with significantly enlarged FBC observed for the same target areas (Figure 9) (see Table S2 for clusterwise FBC values for both cohorts). Generally, FBC appeared to be elevated in the HCP data set. Statistical testing of the most relevant anatomical and functional target regions yielded similar outcomes (Figure 10, Tables S3, S4, and S6). While cluster‐target affiliations were largely replicated in the HCP data set, effect sizes between clusters for a specific target were generally smaller compared to the 1000BRAINS cohort, where more target areas exhibited unique connectivity with only one specific cluster.

Overall, the majority of results were replicable across cohorts, further validating the hypothesis that connectivity patterns of the human insula reflect distinct groups of microstructural areas.

### 3.3 Insula Connectome Analysis in a Whole‐Brain Connectivity Framework

To further investigate the insula's role as a functional integrative hub, we examined its connectome within a comprehensive whole‐brain structural connectivity framework, employing UMAP dimensionality reduction and integrative connectivity maps (see Section 2 for details).

**FIGURE 11:**
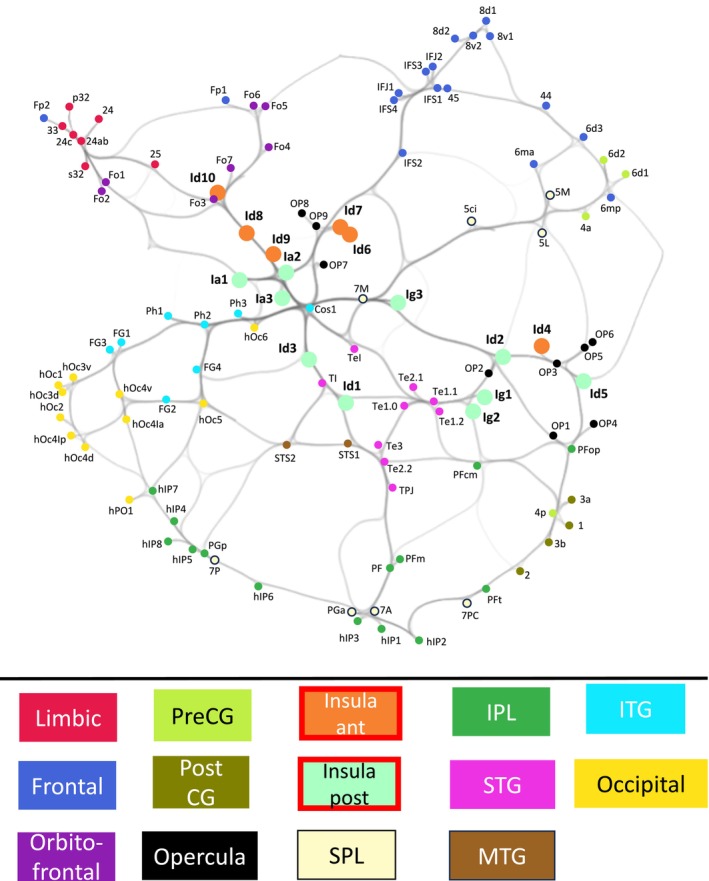
Uniform Manifold Approximation and Projection (UMAP) of the cytoarchitectonic whole‐brain structural connectome. To compare the structural connectome of the insula with the rest of the brain, we used UMAP to generate a two‐dimensional embedding of microstructural connectivity profiles across the entire JulichBrain Atlas. The weighted edges between data points capture local relationships, reflecting proximity in the original feature space in the topological graph structure (e.g., thicker edges indicate stronger similarity between the connectivity profiles of two areas). Brain areas were color‐coded according to their corresponding macroanatomical regions for clarity. Notably, the anterior insula (orange) and the posterior insula (light green) areas emerge as a central hub at the convergence of all major anatomical systems—frontal, parietal, limbic, occipital, and temporal. IPL = inferior parietal lobe; Insula ant = insula anterior; Insula post = insula posterior; ITG = inferior temporal gyrus; MTG = middle temporal gyrus; PostCG = postcentral gyrus; PreCG = precentral gyrus; SPL = superior parietal lobe; STG = superior temporal gyrus.

The UMAP projections (Figure 11) reveal that insular areas, alongside adjacent opercular areas, form a central integrative hub linking frontal, limbic, parietal, temporal, and occipital regions. Connectivity profiles of anterior insular areas more closely resembled those of frontal and limbic regions, whereas posterior insular areas demonstrated stronger alignment with temporal, parietal, and occipital regions. Notably, area Id4 presented an exception: despite its anterior insular anatomical location, its connectivity profile resembled that of posterior insular areas. Area Id10, located within the ventral anterior insula, showed the strongest alignment with orbitofrontal and limbic areas among all insular areas. Both observations were consistent with our findings presented in the cluster analysis.

**FIGURE 12:**
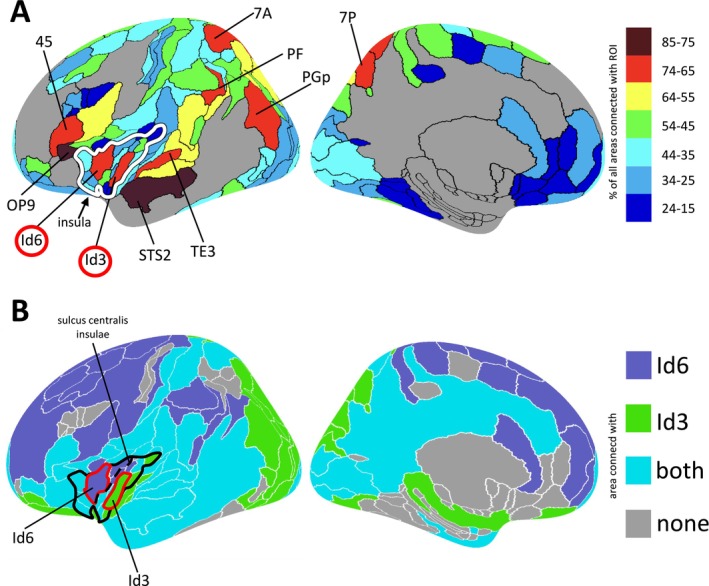
Whole‐brain integrative connectivity profiles. (A) The integrative character of each JulichBrain area was assessed by quantifying the extent of broadly distributed, limited‐strength connections, based on the assumption that integrative areas may not exhibit a few strong connections but instead maintain weaker connections with a wide range of brain areas. Specifically, a connection between two areas was considered to be of “limited strength” if it ranked within the top 50% of the target area's connectivity profile, excluding the strongest connections as determined by a bootstrapped threshold for each target area. Results were projected onto the cortical surface and visualized using the NIH color scheme, ranging from blue to red. For example, a light red ROI indicates that the respective ROI showed limited‐strength connections with approximately 65%–74% of all other areas in the Julich‐Brain Atlas. Two insular areas—Id6 (anterior) and Id3 (posterior)—emerged as among the most widely connected, suggesting an integrative role. (B) *Integrative connectivity profiles of the insular areas Id6 and Id3 were projected onto the cortical surface*. Areas connected exclusively to Id6 are shown in blue, those specific to Id3 in green, areas shared by both in turquoise, and regions with no significant connectivity in gray. Area‐specific differences can be observed, particularly within the frontal and occipital lobes, the orbitofrontal cortex, and the primary auditory cortex. Together with findings from (A), which demonstrated a broad integrative connectivity profile for both areas, these results suggest divergent functional roles. Id6, with its exclusive connectivity to frontal areas, may be involved in higher cognitive integration, whereas Id3 exhibits more targeted connections with specific primary sensory cortices.

The integrative connectivity maps (Figure 12A) further highlighted two insular areas—Id6 and Id3—as among the most extensively connected nodes in the whole‐brain connectome. While both areas shared connectivity with regions of the frontal, parietal, and temporal lobes, they also exhibited clear distinctions (Figure 12B). Specifically, Id6 showed prominent connectivity with most frontal and inferior parietal areas, whereas Id3 displayed exclusive connections with most areas of the occipital lobe, the orbitofrontal cortex, and primary auditory areas within the temporal lobe.

Taken together, the UMAP and integrative connectivity analyses demonstrated that Id6 and Id3 were not only highly connected nodes within the brain‐wide connectome but also uniquely positioned within a central hub in between global connectivity networks (Figure 11). Although other highly connected areas exist within the frontal (areas 45 and OP9), parietal (areas 7A, 7P, PGp, and PF), and temporal lobes (areas STS2 and TE3), only Id6, Id3, and the adjacent opercular area OP9 are located at the intersection of all major anatomical/functional systems—frontal, parietal, temporal, occipital, and limbic (Figure 11).

## 4 Discussion

Using diffusion imaging and tractography in combination with a cytoarchitectonic atlas, this study uncovers unique connectivity patterns in the human insula based on its microstructural architecture. We discovered six distinct clusters that expressed specific connectivity for numerous anatomical and functional target areas. Whole‐brain dimensionality reduction of structural connectivity, along with integrative connectivity maps, demonstrated that insular areas are situated at the intersection of all major brain systems—frontal, parietal, temporal, occipital, and limbic. In particular, insular areas Id6 and Id3 emerged as some of the most extensively connected hubs in the brain, suggesting a central role in integrative processing. These findings advance our understanding of how microstructural architecture shapes structural connectivity in the human insula and provide new insights regarding its integrative function from a whole‐brain structural connectome perspective.

### 4.1 Relationship Between Microstructure and Structural Connectivity in the Human Insula

Our investigation, for the first time, directly assessed the underlying structural connectivity of microstructural areas of the human insula (Figure 2). The results demonstrate no distinct connectivity pattern for each specific area. Recent imaging studies have also indicated a gradual architecture of connectivity within the human insula rather than distinct borders between different connectivity patterns (Cerliani et al. 2012; Royer et al. 2020). These gradients predominantly develop in a posterior‐to‐anterior direction, consistent with our results. Similarly, tracer‐injection studies in the macaque insula have reported a gradual organization of connectivity following the microstructural principle of the granular shift (Amaral and Price 1984; Chikama et al. 1997; Friedman et al. 1986; Fudge et al. 2005). Regions with high (granular) or low (agranular) density of Layer 4 exhibited strong connectivity to target areas with similar granularity, while dysgranular areas showed no such preference (Mesulam and Mufson 1985). Our study corroborates these findings for granular and dysgranular areas. Granular cluster Ig1/Ig2 in the posterior superior insula showed the strongest connectivity with granular opercular areas OP1 and OP2 as well as with the granular primary auditory and somatosensory cortices. By contrast, dysgranular clusters such as, for example, Id4/Id5 showed extensive connectivity across all types of granularity, including agranular motor cortices, granular area OP5, and dysgranular area OP6. Agranular areas, however, did not exhibit specific connectivity patterns in our analysis. The differences might be explained due to the relatively small size of agranular areas, for which high‐resolution methods like tracer injection might yield more accurate results compared to MRI tractography.

However, when microstructural areas are grouped using cluster analysis, the gradual architecture of connectivity transforms into clearly distinguishable patterns between clusters, as well as between the anterior and posterior insula. This finding reflects general microstructural principles of the human insula in two ways. First, our recent study has identified the sulcus centralis insulae as an important cytoarchitectonic landmark, mirroring an overarching change of density in supra‐ and infragranular layers (Quabs et al. 2022). This clear anterior/posterior insula separation has been confirmed in our data and has also been demonstrated in other tractography studies (Jakab et al. 2012; Cloutman et al. 2012). Second, consistent with observations in the macaque insula (Evrard 2019), the human insula's dysgranular field is not homogenous as previously assumed (Mesulam and Mufson 1985); rather, it divides into several distinct dysgranular areas (Quabs et al. 2022). Our data support this differentiation, showing a clear separation in connectivity among dysgranular clusters of the inferior posterior, dorsal anterior, and ventral anterior insula.

Clearly delineated connectivity patterns have also been observed when combining structural and functional connectivity. Recent studies identified three distinct clusters in the posterior, dorsal anterior, and ventral anterior insula (Deen et al. 2011; Nomi et al. 2018; Klugah‐Brown et al. 2023). This separation between anterior/posterior and dorsal/ventral anterior insula aligns with our results. However, combining cytoarchitecture and structural connectivity presents initial evidence to further subdivide the posterior insula into two segments and the dorsal anterior insula into three segments. Especially, the dorsal anterior cluster 1 exhibits a unique bridging profile between the anterior and posterior insula and should be distinguished from other dysgranular clusters in the dorsal anterior insula. Kelly et al. (2012) also demonstrated a possible *k* = 6 cluster solution using a multimodal approach. Their clustering solution showed a similar topographical localization of clusters with two segments in the posterior and four segments in the dorsal anterior insula, supporting a further subdivision of these regions.

The clusters identified in this study exhibited particularly clear differences in connectivity for certain anatomical and functional target regions. These include areas of the orbitofrontal cortex, areas of the frontal and parietal cortices, the planum temporale, as well as frontal networks such as Broca's area, visual, auditory, and somatosensory systems (Figures 6 and 7).

A connection between the insular cortex and the orbitofrontal cortex has been shown in both tracer (R. Augustine 1996; Reiten et al. 2023; Mathiasen et al. 2023) and tractography studies (Ghaziri et al. 2017; Nomi et al. 2018), yet a correlation with a distinct microstructural cluster in the ventral anterior insula has never been described. Given the functional architecture of the ventral anterior insula and the orbitofrontal cortex, the connectivity between both structures may contribute to a network of pathways governing decision‐making and outcome prediction (Droutman et al. 2015; Wang et al. 2023).

Connectivity between the insula and the opercula has been demonstrated in animal studies (R. Augustine 1996), tractography studies (Cerliani et al. 2012; Ghaziri et al. 2017), and particularly in a human dissection study (Demirtaş et al. 2022). Our results are consistent with those findings, providing additional evidence that connectivity strength between the insula and the opercula is substantially greater than between the insula and other target areas. This suggests a high functional‐structural integration within the insulo‐opercular cortex, with opercular areas potentially serving as primary targets within the insular connectome. These insulo‐opercular fiber tracts can be distinctly subdivided based on the cytoarchitecture of the insula. A detailed examination of these tracts is essential to elucidate the extensive functional integration capacity within the insulo‐opercular cortex, involved, for example, in pain processing (Frot 2003) and food prediction (Huang et al. 2021).

Regarding functional systems, our data suggest two distinct clusters in the posterior insula: one linked with auditory information and the other with visual information. This aligns with previous tractography and functional imaging studies, which propose a crucial role for the insula in integrating visual and auditory perceptions (Salomon et al. 2016; Protas 2018), although anatomical connectivity in macaques has only been demonstrated for auditory regions (Mesulam and Mufson 1985). The topography of our identified auditory and visual clusters corresponds with the functional/structural framework presented by Zhang et al. (2019) for these modalities, as well as with electrophysiologically identified sites for auditory processing (Mazzola et al. 2019; Blenkmann et al. 2019). The auditory‐related cluster in the superior posterior insula also coincides in most cases with insular lesions leading to auditory agnosia (Bamiou et al. 2003, 2006).

Our analyses also revealed significant differences in connectivity between somatosensory and frontal networks. Consistent with tracer (Mesulam and Mufson 1985; Rodgers et al. 2008) and tractography studies (Cerliani et al. 2012; Ghaziri et al. 2017), the somatosensory system was linked with the posterior insula, while the frontal regions were primarily connected with the dorsal anterior insular cortex. The involvement of the insula in language‐related processes (Oh et al. 2014; Woolnough et al. 2019; Grodzinsky et al. 2020) was particularly evident in the fibers projecting to Broca's area in our results. This dichotomous connectivity pattern supports the hypothesis that somatosensory stimuli are primarily processed in the posterior insula, whereas the anterior insula is involved in higher cognitive functions. However, our findings suggest a potential microstructural exception to this common concept. The dorsal anterior cluster 1, including areas Id4 and Id5, exhibited increased connectivity with both frontal and somatosensory networks, indicating a unique role among the detected clusters. Further research is required to determine whether the dorsal anterior cluster 1 may function as an integrative bridge, especially between somatosensory and higher cognitive processes in the insula.

Additionally, all findings were replicated using the HCP data set. In this data set, generally higher FBC values were observed, whereas in the 1000BRAINS cohort, a greater number of target areas could be assigned to a single specific cluster. These differences may be attributed to the HCP data set's younger age range and smaller sample size. Nonetheless, the comparison between the two cohorts revealed highly similar clustering results and connectivity patterns (Figure 9).

In contrast to previous tractography studies of the insula, for example, as conducted by Cerliani et al., we employed hard clustering techniques to analyze our data. The earlier work by Cerliani et al. was based primarily on voxel‐wise tractography, without incorporating microstructural characterization of individual voxels, information that was not yet available at the time. As such, applying clustering methods in that context may have been limited by the unaccounted complexity of insular cytoarchitecture, connectivity, and function. Our study builds upon and extends this previous work by integrating microstructural characterization for each insular voxel. This additional layer of information renders hard clustering methods a helpful approach for capturing differences between connectivity patterns of microstructurally defined areas of the insula.

### 4.2 Structural Connectivity of the Insula From a Whole‐Brain Connectome Perspective

The anterior insular cortex has long been proposed as a central integrative hub in the human brain, involved in cognitive control (Wu et al. 2019), emotional awareness (Gu et al. 2013), salience detection (Uddin 2015), and the sense of self (Scalabrini et al. 2022). While much of the supporting evidence derives from functional neuroimaging, the present study provides a complementary structural connectome perspective. Our findings reveal that two specific areas within the insula, Id6 in the anterior insula and Id3 in the posterior insula, are among the most widely connected areas in the brain (Figure 12). These areas, alongside other insular areas, appear to form a core connectivity hub located at the intersection of all major brain systems (Figure 11).

The widely integrative connectivity profile of area Id6 aligns with a recent large‐scale functional meta‐analysis, which identified this area as a key site of functional convergence within the insula (Kwon et al. 2025). Similarly, Kurth, Zilles, et al. (2010) have described a functionally convergent zone in a comparable anatomical location within the insula. In contrast, the role of Id3 in the posterior insula remains less well defined. Intracranial stimulation of this area has been reported to elicit somatosensory experiences, such as tactile sensations and, in some subjects, thermal or painful perceptions (Duong et al. 2023). This aligns with recent meta‐analytic evidence indicating activation of Id3 during pain processing (Kwon et al. 2025).

A widely accepted hypothesis posits that the insula integrates information along a posterior‐to‐anterior axis (Craig 2009). Our findings support this framework, while suggesting that integration does not occur solely in the anterior insula. Instead, we propose the existence of two distinct integrative hubs in the insula, based on the structural connectome: a possible cross‐functional convergence hub in the anterior insula (Id6), and a second, potentially sensory‐specific hub in the posterior insula (Id3). Id6 exhibits exclusive structural connectivity with the frontal cortex and the inferior parietal lobe, consistent with its proposed role in embedding sensory and emotional information within higher‐order cognitive networks. In contrast, Id3 shows selective connections with visual areas and the primary auditory cortex. Combined with evidence from stimulation and functional studies (Duong et al. 2023; Kwon et al. 2025), reporting a participation in feeling of touch and nociception, this pattern suggests that Id3 may be involved in the early stage integration of somatosensory signals before their possible transmission to anterior insula areas for higher‐level processing.

### 4.3 Limitations

A central limitation of our study lies within the DWI tractography itself. While advanced techniques like HARDI and CSD address challenges such as false negatives and crossing fiber issues, current state‐of‐the‐art tractography algorithms encounter difficulties in effectively addressing false positives (Maier‐Hein et al. 2017), particularly evident in probabilistic fiber tracking (Côté et al. 2013). Thus, it is imperative that the biological plausibility of tracts is not solely reliant on the tractography but is complemented by anatomical or electrophysiological approaches. The connectivity analyzed here between microstructural clusters and primary target areas (Figures 6 and 7) aligns with evidence from previous multimodal studies. Connections between the insula and opercula were also shown in human dissection studies (Demirtaş et al. 2022) and tracer injection experiments in macaques (R. Augustine 1996). Further investigations in rodents and primates revealed tracts towards the orbitofrontal cortex (Mufson and Mesulam 1982; R. Augustine 1996; Mathiasen et al. 2023) as well as auditory (Mesulam and Mufson 1985; R. Augustine 1996), frontal, motor (R. Augustine 1996; Gehrlach et al. 2020), and sensation‐related regions (Mesulam and Mufson 1985; R. Augustine 1996; Gehrlach et al. 2020). Additionally, electrophysiological analyses showed connectivity patterns extending to auditory, visual, language, and sensation domains (Dionisio et al. 2019).

Moreover, tractography may not fully capture the connectivity of very small seeds, such as the agranular insular areas of the current study, potentially yielding disparate findings compared to high‐resolution techniques like tracer studies. In general, the white matter configuration surrounding the insular cortex and the directly adjacent claustrum is complex, posing challenges for tractography algorithms to properly reconstruct the anatomy. Especially adjacent white matter voxels medial to the insula encompass several major fiber pathways that traverse the region without necessarily connecting to the insula itself. As a result, tractography in this area is challenged by a low signal‐to‐noise ratio and, in general, low spatial resolution of tractography measures. Therefore, the results of this study indicate significant connectivity differences between microstructural units of the insula and a range of biologically confirmed connections with functional and anatomical target regions with high connectivity strength, which together with complementary evidence from other studies could serve as a basis for further studying the structure–function relations in the human insular cortex.

## 5 Conclusion

Here, we demonstrated that the connectivity patterns of microstructural areas within the human insula are organized in groups characterized by sharp connectivity differences. The clusters reflect overarching cytoarchitectonic principles in the human insula, while connectivity patterns mirroring area‐specific microstructural organization might be detectable with future high‐resolution tractography techniques. The results suggest that adjacent microstructural areas within the insula exhibit similar connectivity patterns, potentially serving different functions in processing similar types of information, which can be categorized into broader clusters. Each of these clusters exhibits different connectivity with a broad spectrum of anatomical and functional target regions, reflecting the insula's role as a multi‐integrational hub region. The strongest connectivity and the most significant connectivity differences were observed between microstructural clusters of the insula and areas of the surrounding opercula and the planum temporale. This finding not only presents new possibilities for surgeons to study growth patterns and underlying functional impairment of tumors in this brain region but also suggests a particularly close functional integrity of the insulo‐opercular region. This study further revealed two integrative hubs within the global and insular connectome: one located in the anterior insula (Id6), possibly serving as a cross‐functional convergence zone for higher‐order integration, and another in the posterior insula (Id3), potentially involved in early‐stage integration of somatosensory information. All identified clusters are provided in standard MNI space (see Data Availability Statement) and can be used as a cytoarchitectonic/structural connectivity framework to further disentangle insular functions, particularly its role in cross‐network functional integration, and pathologies in this complex region of the human neocortex.

## Author Contributions

**Julian Quabs:** writing – original draft, visualization, formal analysis, investigation, methodology, conceptualization. **Nora Bittner:** formal analysis, methodology; writing – review and editing. **Svenja Caspers:** writing – review and editing, supervision, methodology, project administration.

## Conflicts of Interest

The authors declare no conflicts of interest.

## Supporting information


**Figure S1.** Graphical representation of elbow method to obtain optimal number of clusters for (A) clustering all insula areas in the 1000BRAINS cohort, (B) clustering all insula areas in the HCP data set, and (C) clustering the identified groups from approach (A).

**Figure S2.** Clustering of connectivity groups. Multidimensional scaling results in an optimal solution for *k* = 4 clusters (Figure S1). Posterior insula groups formed a cohesive cluster, whereas anterior insula groups exhibited less uniformity. Connectivity fingerprints for each cluster were depicted in (B).

**Figure S3.** Projection of clustered connectivity groups onto the Julich Brain fsaverage template. The color‐coded representation of areas signifies the predominant cluster with the highest fiber bundle capacity in the respective area. The intensity of coloring reflects the actual strength of connectivity.

**Figure S4.** Tract density maps for all insular connectivity clusters. Tract densities within each voxel are color‐coded, ranging from light red indicating high density to dark red representing lower density.

**Table S1.** Average fiber bundle capacity computed for each combination of insular areas and microstructural target areas for the 1000BRAINS cohort and the HCP data set (right and left hemisphere combined). A red cell color denotes that the corresponding connectivity value exceeds the target area‐specific threshold, thereby indicating significantly increased connectivity strength for that insular area relative to all other insular areas within that particular target area. A green cell color indicates that the connectivity value additionally exceeds the threshold for a large effect size, showing that the connectivity strength is significantly increased not only compared to all other insular areas for this specific target but also compared to all other target areas.

**Table S2.** Average fiber bundle capacity computed for each combination of insular clusters and microstructural target areas for the 1000BRAINS cohort and the HCP data set (right and left hemisphere combined). A red cell color denotes that the corresponding connectivity value exceeds the target area‐specific threshold, thereby indicating significantly increased connectivity strength for that cluster relative to all other clusters within that particular target area. A green cell color indicates that the connectivity value additionally exceeds the threshold for a large effect size, showing that the connectivity strength is significantly increased not only compared to all other clusters for this specific target but also compared to all other target areas.

**Table S3.** Conducting a *t*‐test, we examined the cluster with the highest connectivity strength for each opercular target area against the null hypothesis, positing that the connectivity strength does not differ from all other clusters. For each cluster combination, the analysis included reporting the corresponding effect size and the associated confidence interval. The cluster with the highest connectivity strength for each respective target area was considered significant (red caption) if the confidence interval of the effect size exceeded 0.8 for each cluster comparison.

**Table S4.** Conducting a *t*‐test, we examined the cluster with the highest connectivity strength for each anatomical and functional target region against the null hypothesis, positing that the connectivity strength does not differ from all other clusters. For each cluster combination, the analysis included reporting the corresponding effect size and the associated confidence interval. The cluster with the highest connectivity strength for each target area was considered significant (red caption) if the confidence interval of the effect size exceeded 0.8 for each cluster comparison.

**Table S5.** Average fiber bundle capacity per subject computed for each combination of insular connectivity cluster and functional/anatomical target region for the 1000BRAINS data set (right and left hemisphere combined).

**Table S6.** Average fiber bundle capacity per subject computed for each combination of insular connectivity cluster and functional/anatomical target region for the HCP data set (right and left hemisphere combined).

## Data Availability

All identified clusters in MNI space are publicly available under: https://jugit.fz‐juelich.de/inm‐1/connectivity/release/Insula_microstructure_strutural_connectivity.git.

## References

[hbm70231-bib-0001] Aggarwal, C. C. , A. Hinneburg , and D. A. Keim . 2001. “On the Surprising Behavior of Distance Metrics in High Dimensional Space.” In Database Theory—ICDT 2001, Lecture Notes in Computer Science, edited by J. van Den Bussche and V. Vianu , vol. 1973, 420–434. Springer Berlin Heidelberg. 10.1007/3-540-44503-X_27.

[hbm70231-bib-0002] Amaral, D. G. , and J. L. Price . 1984. “Amygdalo‐Cortical Projections in the Monkey ( *Macaca fascicularis* ).” Journal of Comparative Neurology 230, no. 4: 465–496. 10.1002/cne.902300402. 6520247

[hbm70231-bib-0003] Amunts, K. , H. Mohlberg , S. Bludau , and K. Zilles . 2020. “Julich‐Brain: A 3D Probabilistic Atlas of the Human Brain's Cytoarchitecture.” Science 369, no. 6506: 988–992. 10.1126/science.abb4588. 32732281

[hbm70231-bib-0004] Andersson, J. L. R. , M. S. Graham , E. Zsoldos , and S. N. Sotiropoulos . 2016. “Incorporating Outlier Detection and Replacement Into a Non‐Parametric Framework for Movement and Distortion Correction of Diffusion MR Images.” NeuroImage 141: 556–572. 10.1016/j.neuroimage.2016.06.058. 27393418

[hbm70231-bib-0005] Andersson, J. L. R. , S. Skare , and J. Ashburner . 2003. “How to Correct Susceptibility Distortions in Spin‐Echo Echo‐Planar Images: Application to Diffusion Tensor Imaging.” NeuroImage 20, no. 2: 870–888. 10.1016/S1053-8119(03)00336-7. 14568458

[hbm70231-bib-0006] Andersson, J. L. R. , and S. N. Sotiropoulos . 2015. “Non‐Parametric Representation and Prediction of Single‐ and Multi‐Shell Diffusion‐Weighted MRI Data Using Gaussian Processes.” NeuroImage 122: 166–176. 10.1016/j.neuroimage.2015.07.067. 26236030 PMC4627362

[hbm70231-bib-0007] Augustine, J. R. 1985. “The Insular Lobe in Primates Including Humans.” Neurological Research 7, no. 1: 2–10. 10.1080/01616412.1985.11739692. 2860583

[hbm70231-bib-0008] Augustine, R. 1996. “Circuitry and Fimctional Aspects of the Insular Lobe in Primates Including Humans.” Brain Research Reviews 22, no. 3: 229–244. 8957561 10.1016/s0165-0173(96)00011-2

[hbm70231-bib-0009] Avants, B. B. , N. J. Tustison , M. Stauffer , G. Song , B. Wu , and J. C. Gee . 2014. “The Insight ToolKit Image Registration Framework.” Frontiers in Neuroinformatics 8: 44. 10.3389/fninf.2014.00044. 24817849 PMC4009425

[hbm70231-bib-0010] Bamiou, D. E. , F. E. Musiek , and L. M. Luxon . 2003. “The Insula (Island of Reil) and Its Role in Auditory Processing Literature Review.” Brain Research Reviews 42, no. 2: 143–154. 12738055 10.1016/s0165-0173(03)00172-3

[hbm70231-bib-0011] Bamiou, D. E. , F. E. Musiek , I. Stow , et al. 2006. “Auditory Temporal Processing Deficits in Patients With Insular Stroke.” Neurology 67, no. 4: 614–619. 10.1212/01.wnl.0000230197.40410.db. 16924014

[hbm70231-bib-0012] Blenkmann, A. O. , S. Collavini , J. Lubell , et al. 2019. “Auditory Deviance Detection in the Human Insula: An Intracranial EEG Study.” Cortex 121: 189–200. 10.1016/j.cortex.2019.09.002. 31629197

[hbm70231-bib-0013] Bludau, S. , T. W. Mühleisen , S. B. Eickhoff , M. J. Hawrylycz , S. Cichon , and K. Amunts . 2018. “Integration of Transcriptomic and Cytoarchitectonic Data Implicates a Role for MAOA and TAC1 in the Limbic‐Cortical Network.” Brain Structure & Function 223, no. 5: 2335–2342. 10.1007/s00429-018-1620-6. 29478144 PMC5968065

[hbm70231-bib-0014] Bluma, A. L. , and P. Langley . 1997. “Selection of Relevant Features and Examples in Machine Learning.” Artificial Intelligence 97, no. 1–2: 245–271.

[hbm70231-bib-0015] Craig, A. D. 2009. “How Do You Feel—Now? The Anterior Insula and Human Awareness.” Nature Reviews Neuroscience 10, no. 1: 59–70. 10.1038/nrn2555. 19096369

[hbm70231-bib-0016] Carmichael, S. T. , and J. L. Price . 1995. “Limbic Connections of the Orbital and Medial Prefrontal Cortex in Macaque Monkeys.” Journal of Comparative Neurology 363, no. 4: 615–641. 10.1002/cne.903630408. 8847421

[hbm70231-bib-0017] Caspers, S. , S. B. Eickhoff , K. Zilles , and K. Amunts . 2013. “Microstructural Grey Matter Parcellation and Its Relevance for Connectome Analyses.” NeuroImage 80: 18–26. 10.1016/j.neuroimage.2013.04.003. 23571419 PMC8010271

[hbm70231-bib-0018] Caspers, S. , S. Moebus , S. Lux , et al. 2014. “Studying Variability in Human Brain Aging in a Population‐Based German Cohort—Rationale and Design of 1000BRAINS.” Frontiers in Aging Neuroscience 6: 149. 10.3389/fnagi.2014.00149. 25071558 PMC4094912

[hbm70231-bib-0019] Caspers, S. , and K. Zilles . 2018. “Microarchitecture and Connectivity of the Parietal Lobe.” In Handbook of Clinical Neurology, 53–72. Elsevier. 10.1016/B978-0-444-63622-5.00003-6151. 29519479

[hbm70231-bib-0020] Cerliani, L. , R. M. Thomas , S. Jbabdi , et al. 2012. “Probabilistic Tractography Recovers a Rostrocaudal Trajectory of Connectivity Variability in the Human Insular Cortex.” Human Brain Mapping 33, no. 9: 2005–2034. 10.1002/hbm.21338. 21761507 PMC3443376

[hbm70231-bib-0021] Chikama, M. , N. R. McFarland , D. G. Amaral , and S. N. Haber . 1997. “Insular Cortical Projections to Functional Regions of the Striatum Correlate With Cortical Cytoarchitectonic Organization in the Primate.” Journal of Neuroscience 17, no. 24: 9686–9705. 10.1523/JNEUROSCI.17-24-09686.1997. 9391023 PMC6573402

[hbm70231-bib-0022] Civier, O. , R. E. Smith , C. H. Yeh , A. Connelly , and F. Calamante . 2019. “Is Removal of Weak Connections Necessary for Graph‐Theoretical Analysis of Dense Weighted Structural Connectomes From Diffusion MRI?” NeuroImage 194: 68–81. 10.1016/j.neuroimage.2019.02.039. 30844506

[hbm70231-bib-0023] Cloutman, L. L. , R. J. Binney , M. Drakesmith , G. J. M. Parker , and M. A. Lambon Ralph . 2012. “The Variation of Function Across the Human Insula Mirrors Its Patterns of Structural Connectivity: Evidence From In Vivo Probabilistic Tractography.” NeuroImage 59, no. 4: 3514–3521. 10.1016/j.neuroimage.2011.11.016. 22100771

[hbm70231-bib-0024] Côté, M. A. , G. Girard , A. Boré , E. Garyfallidis , J. C. Houde , and M. Descoteaux . 2013. “Tractometer: Towards Validation of Tractography Pipelines.” Medical Image Analysis 17, no. 7: 844–857. 10.1016/j.media.2013.03.009. 23706753

[hbm70231-bib-0025] Craig, A. D. 2003. “Interoception: The Sense of the Physiological Condition of the Body.” Current Opinion in Neurobiology 13, no. 4: 500–505. 10.1016/S0959-4388(03)00090-4. 12965300

[hbm70231-bib-0026] Dale, A. M. , B. Fischl , and M. I. Sereno . 1999. “Cortical Surface‐Based Analysis. I. Segmentation and Surface Reconstruction.” NeuroImage 9, no. 2: 179–194. 9931268 10.1006/nimg.1998.0395

[hbm70231-bib-0027] Deen, B. , N. B. Pitskel , and K. A. Pelphrey . 2011. “Three Systems of Insular Functional Connectivity Identified With Cluster Analysis.” Cerebral Cortex 21, no. 7: 1498–1506. 10.1093/cercor/bhq186. 21097516 PMC3116731

[hbm70231-bib-0028] Dell'Acqua, F. , L. M. Lacerda , M. Catani , and A. Simmons . 2013. “Anisotropic Power Maps: A Diffusion Contrast to Reveal Low Anisotropy Tissues From HARDI Data.” https://api.semanticscholar.org/CorpusID:46663779.

[hbm70231-bib-0029] Demirtaş, O. K. , A. Güngör , P. Çeltikçi , et al. 2022. “Microsurgical Anatomy and Insular Connectivity of the Cerebral Opercula.” Journal of Neurosurgery 137, no. 5: 1509–1523. 10.3171/2021.12.JNS212297. 35303697

[hbm70231-bib-0030] Denis, D. J. , R. Marouf , P. Rainville , A. Bouthillier , and D. K. Nguyen . 2016. “Effects of Insular Stimulation on Thermal Nociception.” European Journal of Pain 20, no. 5: 800–810. 10.1002/ejp.806. 26471114

[hbm70231-bib-0031] Dhollander, T. , A. Clemente , M. Singh , et al. 2021. “Fixel‐Based Analysis of Diffusion MRI: Methods, Applications, Challenges and Opportunities.” NeuroImage 241: 118417. 10.1016/j.neuroimage.2021.118417. 34298083

[hbm70231-bib-0032] Dionisio, S. , L. Mayoglou , S. M. Cho , et al. 2019. “Connectivity of the Human Insula: A Cortico‐Cortical Evoked Potential (CCEP) Study.” Cortex 120: 419–442. 10.1016/j.cortex.2019.05.019. 31442863 PMC6825888

[hbm70231-bib-0033] Droutman, V. , A. Bechara , and S. J. Read . 2015. “Roles of the Different Sub‐Regions of the Insular Cortex in Various Phases of the Decision‐Making Process.” Frontiers in Behavioral Neuroscience 9: 309. 10.3389/fnbeh.2015.00309. 26635559 PMC4658437

[hbm70231-bib-0034] Duong, A. , J. Quabs , A. Kucyi , et al. 2023. “Subjective States Induced by Intracranial Electrical Stimulation Matches the Cytoarchitectonic Organization of the Human Insula.” Brain Stimulation 16, no. 6: 1653–1665. 10.1016/j.brs.2023.11.001. 37949296 PMC10893903

[hbm70231-bib-0035] Eickhoff, S. B. , S. Jbabdi , S. Caspers , et al. 2010. “Anatomical and Functional Connectivity of Cytoarchitectonic Areas Within the Human Parietal Operculum.” Journal of Neuroscience 30, no. 18: 6409–6421. 10.1523/JNEUROSCI.5664-09.2010. 20445067 PMC4791040

[hbm70231-bib-0036] Erbel, R. , L. Eisele , S. Moebus , et al. 2012. “Die Heinz Nixdorf Recall Studie.” Bundesgesundheitsbl 55, no. 6–7: 809–815. 10.1007/s00103-012-1490-7. 22736160

[hbm70231-bib-0037] Evrard, H. C. 2019. “The Organization of the Primate Insular Cortex.” Frontiers in Neuroanatomy 13: 43. 10.3389/fnana.2019.00043. 31133822 PMC6517547

[hbm70231-bib-0038] Evrard, H. C. , N. K. Logothetis , and A. D. (Bud) Craig . 2014. “Modular Architectonic Organization of the Insula in the Macaque Monkey.” Journal of Comparative Neurology 522, no. 1: 64–97. 10.1002/cne.23436. 23900781

[hbm70231-bib-0039] Fischl, B. 2012. “FreeSurfer.” NeuroImage 62, no. 2: 774–781. 10.1016/j.neuroimage.2012.01.021. 22248573 PMC3685476

[hbm70231-bib-0040] Friedman, D. P. , E. A. Murray , J. B. O'Neill , and M. Mishkin . 1986. “Cortical Connections of the Somatosensory Fields of the Lateral Sulcus of Macaques: Evidence for a Corticolimbic Pathway for Touch.” Journal of Comparative Neurology 252, no. 3: 323–347. 10.1002/cne.902520304. 3793980

[hbm70231-bib-0041] Frot, M. 2003. “Dual Representation of Pain in the Operculo‐Insular Cortex in Humans.” Brain 126, no. 2: 438–450. 10.1093/brain/awg032. 12538410

[hbm70231-bib-0042] Fu, Y. , Z. Long , Q. Luo , et al. 2021. “Functional and Structural Connectivity Between the Left Dorsolateral Prefrontal Cortex and Insula Could Predict the Antidepressant Effects of Repetitive Transcranial Magnetic Stimulation.” Frontiers in Neuroscience 15: 645936. 10.3389/fnins.2021.645936. 33841087 PMC8032871

[hbm70231-bib-0043] Fudge, J. L. , M. A. Breitbart , M. Danish , and V. Pannoni . 2005. “Insular and Gustatory Inputs to the Caudal Ventral Striatum in Primates.” Journal of Comparative Neurology 490, no. 2: 101–118. 10.1002/cne.20660. 16052493 PMC2474655

[hbm70231-bib-0044] Gaser, C. , R. Dahnke , P. M. Thompson , F. Kurth , E. Luders , and Alzheimer's Disease Neuroimaging Initiative . 2022. “CAT—A Computational Anatomy Toolbox for the Analysis of Structural MRI Data.” Published Online June 13. 10.1101/2022.06.11.495736. PMC11299546 39102518

[hbm70231-bib-0045] Gehrlach, D. A. , C. Weiand , T. N. Gaitanos , et al. 2020. “A Whole‐Brain Connectivity Map of Mouse Insular Cortex.” eLife 9: e55585. 10.7554/eLife.55585. 32940600 PMC7538160

[hbm70231-bib-0046] Gere, A. 2023. “Recommendations for Validating Hierarchical Clustering in Consumer Sensory Projects.” Current Research in Food Science 6: 100522. 10.1016/j.crfs.2023.100522. 37266412 PMC10230197

[hbm70231-bib-0047] Ghaziri, J. , A. Tucholka , G. Girard , et al. 2018. “Subcortical Structural Connectivity of Insular Subregions.” Scientific Reports 8, no. 1: 8596. 10.1038/s41598-018-26995-0. 29872212 PMC5988839

[hbm70231-bib-0048] Ghaziri, J. , A. Tucholka , G. Girard , et al. 2017. “The Corticocortical Structural Connectivity of the Human Insula.” Cerebral Cortex 27, no. 2: 1216–1228. 10.1093/cercor/bhv308. 26683170

[hbm70231-bib-0049] Goodkind, M. , S. B. Eickhoff , D. J. Oathes , et al. 2015. “Identification of a Common Neurobiological Substrate for Mental Illness.” JAMA Psychiatry 72, no. 4: 305–315. 10.1001/jamapsychiatry.2014.2206. 25651064 PMC4791058

[hbm70231-bib-0050] Grodzinsky, Y. , I. Deschamps , P. Pieperhoff , et al. 2020. “Logical Negation Mapped Onto the Brain.” Brain Structure & Function 225, no. 1: 19–31. 10.1007/s00429-019-01975-w. 31680213 PMC6957563

[hbm70231-bib-0051] Gu, X. , P. R. Hof , K. J. Friston , and J. Fan . 2013. “Anterior Insular Cortex and Emotional Awareness: Anterior Insular Cortex and Emotional Awareness.” Journal of Comparative Neurology 521, no. 15: 3371–3388. 10.1002/cne.23368. 23749500 PMC3999437

[hbm70231-bib-0052] Guevara, M. , C. Román , J. Houenou , et al. 2017. “Reproducibility of Superficial White Matter Tracts Using Diffusion‐Weighted Imaging Tractography.” NeuroImage 147: 703–725. 10.1016/j.neuroimage.2016.11.066. 28034765

[hbm70231-bib-0053] Guevara, P. , D. Duclap , C. Poupon , et al. 2012. “Automatic Fiber Bundle Segmentation in Massive Tractography Datasets Using a Multi‐Subject Bundle Atlas.” NeuroImage 61, no. 4: 1083–1099. 10.1016/j.neuroimage.2012.02.071. 22414992

[hbm70231-bib-0054] Hassanpour, M. S. , W. K. Simmons , J. S. Feinstein , et al. 2018. “The Insular Cortex Dynamically Maps Changes in Cardiorespiratory Interoception.” Neuropsychopharmacology 43, no. 2: 426–434. 10.1038/npp.2017.154. 28726799 PMC5729563

[hbm70231-bib-0055] Hein, M. n.d. “Probabilistic Cytoarchitectonic Map of Area Ia2, Ia3, Id8, Id9, Id10.”

[hbm70231-bib-0056] Huang, Y. , B. W. Kakusa , A. Feng , et al. 2021. “The Insulo‐Opercular Cortex Encodes Food‐Specific Content Under Controlled and Naturalistic Conditions.” Nature Communications 12, no. 1: 3609. 10.1038/s41467-021-23885-4. PMC8203663 34127675

[hbm70231-bib-0057] Jakab, A. , P. P. Molnár , P. Bogner , M. Béres , and E. L. Berényi . 2012. “Connectivity‐Based Parcellation Reveals Interhemispheric Differences in the Insula.” Brain Topography 25, no. 3: 264–271. 10.1007/s10548-011-0205-y. 22002490

[hbm70231-bib-0058] Jarman, A. M. 2020. “Hierarchical Cluster Analysis: Comparison of Single Linkage, Complete Linkage, Average Linkage and Centroid Linkage Method.” 10.13140/RG.2.2.11388.90240.

[hbm70231-bib-0059] Jenkinson, M. , C. F. Beckmann , T. E. J. Behrens , M. W. Woolrich , and S. M. Smith . 2012. “FSL.” NeuroImage 62, no. 2: 782–790. 10.1016/j.neuroimage.2011.09.015 FSL. 21979382

[hbm70231-bib-0060] Kelly, C. , R. Toro , A. Di Martino , et al. 2012. “A Convergent Functional Architecture of the Insula Emerges Across Imaging Modalities.” NeuroImage 61, no. 4: 1129–1142. 10.1016/j.neuroimage.2012.03.021. 22440648 PMC3376229

[hbm70231-bib-0061] Khalsa, S. S. , R. Adolphs , O. G. Cameron , et al. 2018. “Interoception and Mental Health: A Roadmap.” Biological Psychiatry: Cognitive Neuroscience and Neuroimaging 3, no. 6: 501–513. 10.1016/j.bpsc.2017.12.004. 29884281 PMC6054486

[hbm70231-bib-0062] Klepzig, K. , M. Domin , J. Wendt , et al. 2023. “Structural Integrity of the Insula and Emotional Facial Recognition Performance Following Stroke.” Brain Communications 5, no. 3: fcad144. 10.1093/braincomms/fcad144. 37292458 PMC10244053

[hbm70231-bib-0063] Klugah‐Brown, B. , P. Wang , Y. Jiang , et al. 2023. “Structural–Functional Connectivity Mapping of the Insular Cortex: A Combined Data‐Driven and Meta‐Analytic Topic Mapping.” Cerebral Cortex 33, no. 5: 1726–1738. 10.1093/cercor/bhac168. 35511500

[hbm70231-bib-0064] Kurth, F. , S. B. Eickhoff , A. Schleicher , K. Hoemke , K. Zilles , and K. Amunts . 2010. “Cytoarchitecture and Probabilistic Maps of the Human Posterior Insular Cortex.” Cerebral Cortex 20, no. 6: 1448–1461. 10.1093/cercor/bhp208. 19822572 PMC2871375

[hbm70231-bib-0065] Kurth, F. , K. Zilles , P. T. Fox , A. R. Laird , and S. B. Eickhoff . 2010. “A Link Between the Systems: Functional Differentiation and Integration Within the Human Insula Revealed by Meta‐Analysis.” Brain Structure & Function 214, no. 5–6: 519–534. 10.1007/s00429-010-0255-z. 20512376 PMC4801482

[hbm70231-bib-0066] Kwon, M. , K. Bo , R. Botvinik‐Nezer , et al. 2025. “Convergent and Selective Representations of Pain, Appetitive Processes, Aversive Processes, and Cognitive Control in the Insula.” Published Online February 19. 10.1101/2025.02.18.638889. PMC13254212 41980935

[hbm70231-bib-0067] Lin, M. , H. C. Lucas , and G. Shmueli . 2013. “Research Commentary—Too Big to Fail: Large Samples and the *p*‐Value Problem.” Information Systems Research 24, no. 4: 906–917. 10.1287/isre.2013.0480.

[hbm70231-bib-0068] Lotze, M. 2024. “Emotional Processing Impairments in Patients With Insula Lesions Following Stroke.” NeuroImage 291: 120591. 10.1016/j.neuroimage.2024.120591. 38552812

[hbm70231-bib-0069] Maier‐Hein, K. H. , P. F. Neher , J. C. Houde , et al. 2017. “The Challenge of Mapping the Human Connectome Based on Diffusion Tractography.” Nature Communications 8, no. 1: 1349. 10.1038/s41467-017-01285-x. PMC5677006 29116093

[hbm70231-bib-0070] Mathiasen, M. L. , J. P. Aggleton , and M. P. Witter . 2023. “Projections of the Insular Cortex to Orbitofrontal and Medial Prefrontal Cortex: A Tracing Study in the Rat.” Frontiers in Neuroanatomy 17: 1131167. 10.3389/fnana.2023.1131167. 37152205 PMC10158940

[hbm70231-bib-0071] Mazzola, L. , C. Lopez , I. Faillenot , F. Chouchou , F. Mauguière , and J. Isnard . 2014. “Vestibular Responses to Direct Stimulation of the Human Insular Cortex: Insula Stimulation Response.” Annals of Neurology 76, no. 4: 609–619. 10.1002/ana.24252. 25142204

[hbm70231-bib-0072] Mazzola, L. , F. Mauguière , and J. Isnard . 2019. “Functional Mapping of the Human Insula: Data From Electrical Stimulations.” Revue Neurologique 175, no. 3: 150–156. 10.1016/j.neurol.2018.12.003. 30827578

[hbm70231-bib-0073] McInnes, L. , J. Healy , and J. Melville . 2020. “UMAP: Uniform Manifold Approximation and Projection for Dimension Reduction.” Published Online September 18. 10.48550/arXiv.1802.03426.

[hbm70231-bib-0074] Mesulam, M. M. , and E. J. Mufson . 1982. “Insula of the Old World Monkey. III: Efferent Cortical Output and Comments on Function.” Journal of Comparative Neurology 212, no. 1: 38–52. 10.1002/cne.902120104. 7174907

[hbm70231-bib-0075] Mesulam, M. M. , and E. J. Mufson . 1982. “Insula of the Old World Monkey. Architectonics in the Insulo‐Orbito‐Temporal Component of the Paralimbic Brain.” Journal of Comparative Neurology 212, no. 1: 1–22. 10.1002/cne.902120102. 7174905

[hbm70231-bib-0076] Mesulam, M. M. , and E. J. Mufson . 1985. “The Insula of Reil in Man and Monkey.” In Association and Auditory Cortices, edited by A. Peters and E. G. Jones , vol. 4, 179–226. Springer US. 10.1007/978-1-4757-9619-3_5.

[hbm70231-bib-0077] Mufson, E. J. , and M. M. Mesulam . 1982. “Insula of the Old World Monkey. II: Afferent Cortical Input and Comments on the Claustrum.” Journal of Comparative Neurology 212, no. 1: 23–37. 10.1002/cne.902120103. 7174906

[hbm70231-bib-0078] Mufson, E. J. , and M. M. Mesulam . 1984. “Thalamic Connections of the Insula in the Rhesus Monkey and Comments on the Paralimbic Connectivity of the Medial Pulvinar Nucleus.” Journal of Comparative Neurology 227, no. 1: 109–120. 10.1002/cne.902270112. 6470205

[hbm70231-bib-0079] Mufson, E. J. , M. M. Mesulam , and D. N. Pandya . 1981. “Insular Interconnections With the Amygdala in the Rhesus Monkey.” Neuroscience 6, no. 7: 1231–1248. 10.1016/0306-4522(81)90184-6. 6167896

[hbm70231-bib-0080] Mugler, J. P. , and J. R. Brookeman . 1990. “Three‐Dimensional Magnetization‐Prepared Rapid Gradient‐Echo Imaging (3D MP RAGE).” Magnetic Resonance in Medicine 15, no. 1: 152–157. 10.1002/mrm.1910150117. 2374495

[hbm70231-bib-0081] Nomi, J. S. , E. Schettini , I. Broce , A. S. Dick , and L. Q. Uddin . 2018. “Structural Connections of Functionally Defined Human Insular Subdivisions.” Cerebral Cortex 28, no. 10: 3445–3456. 10.1093/cercor/bhx211. 28968768 PMC6132280

[hbm70231-bib-0082] Nord, C. L. , R. P. Lawson , and T. Dalgleish . 2021. “Disrupted Dorsal Mid‐Insula Activation During Interoception Across Psychiatric Disorders.” American Journal of Psychiatry 178, no. 8: 761–770. 10.1176/appi.ajp.2020.20091340. 34154372 PMC7613124

[hbm70231-bib-0083] Obaid, S. , F. Rheault , M. Edde , et al. 2021. “Structural Connectivity Alterations in Operculo‐Insular Epilepsy.” Brain Sciences 11, no. 8: 1041. 10.3390/brainsci11081041. 34439659 PMC8392362

[hbm70231-bib-0084] Oh, A. , E. G. Duerden , and E. W. Pang . 2014. “The Role of the Insula in Speech and Language Processing.” Brain and Language 135: 96–103. 10.1016/j.bandl.2014.06.003. 25016092 PMC4885738

[hbm70231-bib-0085] Protas, M. 2018. “Role of the Insula in Visual and Auditory Perception.” In Island of Reil (Insula) in the Human Brain: Anatomical, Functional, Clinical and Surgical Aspects, edited by M. Turgut , C. Yurttaş , and R. S. Tubbs , 151–156. Springer International Publishing. 10.1007/978-3-319-75468-0_16.

[hbm70231-bib-0086] Quabs, J. , S. Caspers , C. Schöne , et al. 2022. “Cytoarchitecture, Probability Maps and Segregation of the Human Insula.” NeuroImage 260: 119453. 10.1016/j.neuroimage.2022.119453. 35809885

[hbm70231-bib-0087] Reiten, I. , G. M. Olsen , J. G. Bjaalie , M. P. Witter , and T. B. Leergaard . 2023. “The Efferent Connections of the Orbitofrontal, Posterior Parietal, and Insular Cortex of the Rat Brain.” Scientific Data 10, no. 1: 645. 10.1038/s41597-023-02527-y. 37735463 PMC10514078

[hbm70231-bib-0088] Rodgers, K. M. , A. M. Benison , A. Klein , and D. S. Barth . 2008. “Auditory, Somatosensory, and Multisensory Insular Cortex in the Rat.” Cerebral Cortex 18, no. 12: 2941–2951. 10.1093/cercor/bhn054. 18424777 PMC2583160

[hbm70231-bib-0089] Royer, J. , C. Paquola , S. Larivière , et al. 2020. “Myeloarchitecture Gradients in the Human Insula: Histological Underpinnings and Association to Intrinsic Functional Connectivity.” NeuroImage 216: 116859. 10.1016/j.neuroimage.2020.116859. 32325211

[hbm70231-bib-0090] Salomon, R. , R. Ronchi , J. Dönz , et al. 2016. “The Insula Mediates Access to Awareness of Visual Stimuli Presented Synchronously to the Heartbeat.” Journal of Neuroscience 36, no. 18: 5115–5127. 10.1523/JNEUROSCI.4262-15.2016. 27147663 PMC6601849

[hbm70231-bib-0091] Scalabrini, A. , C. Mucci , and G. Northoff . 2022. “The Nested Hierarchy of Self and Its Trauma: In Search for a Synchronic Dynamic and Topographical Re‐Organization.” Frontiers in Human Neuroscience 16: 980353. 10.3389/fnhum.2022.980353. 36118976 PMC9478193

[hbm70231-bib-0092] Schmermund, A. , S. Möhlenkamp , A. Stang , et al. 2002. “Assessment of Clinically Silent Atherosclerotic Disease and Established and Novel Risk Factors for Predicting Myocardial Infarction and Cardiac Death in Healthy Middle‐Aged Subjects: Rationale and Design of the Heinz Nixdorf RECALL Study.” American Heart Journal 144, no. 2: 212–218. 10.1067/mhj.2002.123579. 12177636

[hbm70231-bib-0093] Smith, R. E. 2020. “Quantitative Streamlines Tractography: Methods and Inter‐Subject Normalisation.”

[hbm70231-bib-0094] Smith, R. E. , J. D. Tournier , F. Calamante , and A. Connelly . 2012. “Anatomically‐Constrained Tractography: Improved Diffusion MRI Streamlines Tractography Through Effective Use of Anatomical Information.” NeuroImage 62, no. 3: 1924–1938. 10.1016/j.neuroimage.2012.06.005. 22705374

[hbm70231-bib-0095] Taylor, J. J. , C. Lin , D. Talmasov , et al. 2023. “A Transdiagnostic Network for Psychiatric Illness Derived From Atrophy and Lesions.” Nature Human Behaviour 7, no. 3: 420–429. 10.1038/s41562-022-01501-9. PMC10236501 36635585

[hbm70231-bib-0096] Tournier, J. , F. Calamante , and A. Connelly . 2012. “MRtrix: Diffusion Tractography in Crossing Fiber Regions.” International Journal of Imaging Systems and Technology 22, no. 1: 53–66. 10.1002/ima.22005.

[hbm70231-bib-0097] Uddin, L. Q. 2015. “Salience Processing and Insular Cortical Function and Dysfunction.” Nature Reviews. Neuroscience 16, no. 1: 55–61. 10.1038/nrn3857. 25406711

[hbm70231-bib-0098] Umargono, E. , J. E. Suseno , and S. K. VG . 2019. “K‐Means Clustering Optimization Using the Elbow Method and Early Centroid Determination Based‐On Mean and Median.” In Proceedings of the International Conferences on Information System and Technology, 234–240. SCITEPRESS – Science and Technology Publications. 10.5220/0009908402340240.

[hbm70231-bib-0099] Van Essen, D. C. , S. M. Smith , D. M. Barch , T. E. J. Behrens , E. Yacoub , and K. Ugurbil . 2013. “The WU‐Minn Human Connectome Project: An Overview.” NeuroImage 80: 62–79. 10.1016/j.neuroimage.2013.05.041. 23684880 PMC3724347

[hbm70231-bib-0100] Wakana, S. , A. Caprihan , M. M. Panzenboeck , et al. 2007. “Reproducibility of Quantitative Tractography Methods Applied to Cerebral White Matter.” NeuroImage 36, no. 3: 630–644. 10.1016/j.neuroimage.2007.02.049. 17481925 PMC2350213

[hbm70231-bib-0101] Wang, B. A. , M. Veismann , A. Banerjee , and B. Pleger . 2023. “Human Orbitofrontal Cortex Signals Decision Outcomes to Sensory Cortex During Behavioral Adaptations.” Nature Communications 14, no. 1: 3552. 10.1038/s41467-023-38671-7. PMC10272188 37322004

[hbm70231-bib-0102] Woolnough, O. , K. J. Forseth , P. S. Rollo , and N. Tandon . 2019. “Uncovering the Functional Anatomy of the Human Insula During Speech.” eLife 8: e53086. 10.7554/eLife.53086. 31852580 PMC6941893

[hbm70231-bib-0103] Wu, T. , X. Wang , Q. Wu , et al. 2019. “Anterior Insular Cortex Is a Bottleneck of Cognitive Control.” NeuroImage 195: 490–504. 10.1016/j.neuroimage.2019.02.042. 30798012 PMC6550348

[hbm70231-bib-0104] Zhang, Y. , W. Zhou , S. Wang , et al. 2019. “The Roles of Subdivisions of Human Insula in Emotion Perception and Auditory Processing.” Cerebral Cortex 29, no. 2: 517–528. 10.1093/cercor/bhx334. 29342237

