# Supplementary figures and images for: Structural Connectivity Differences Reflect Microstructural Heterogeneity of the Human Insular Cortex
Source: Hum Brain Mapp. 2025 May 21;46(8):e70231. doi: 10.1002/hbm.70231 (PMC12093499; doi:10.1002/hbm.70231)

A

## 1000BRAINS – all areas

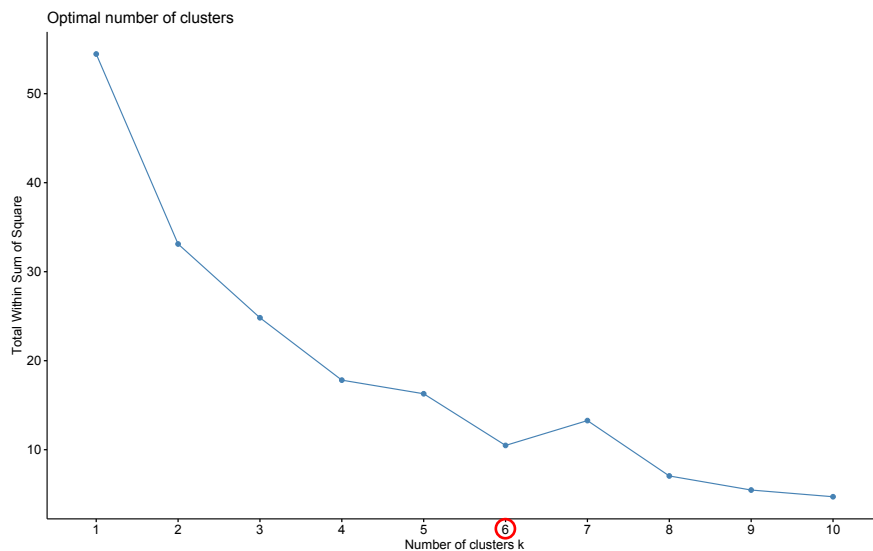

B

## HCP – all areas

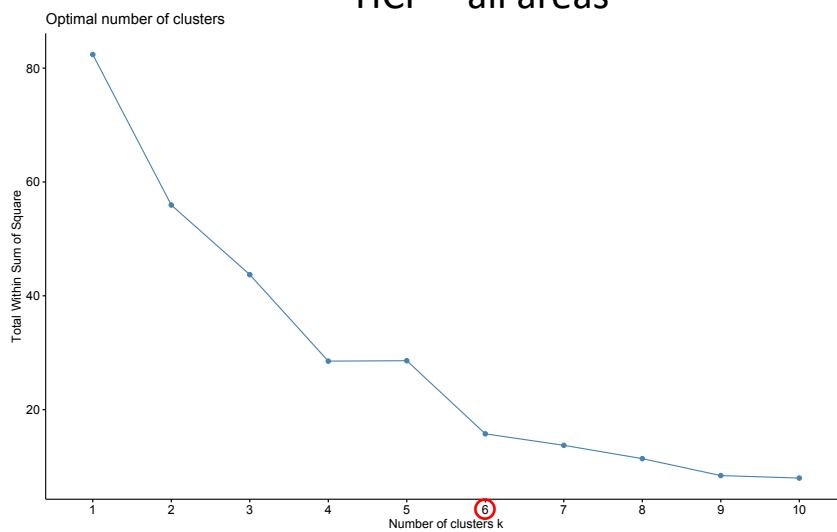

C

## 1000BRAINS – all clusters

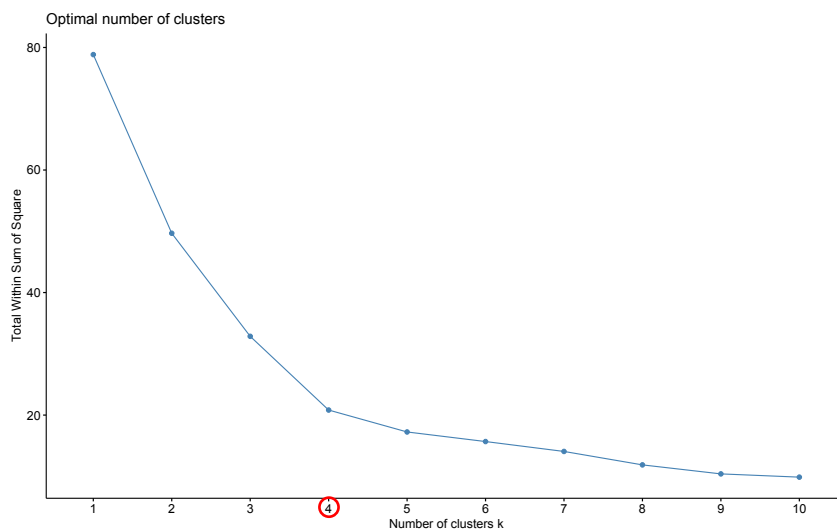

Supplement: Supplementary file 1 — Figure S1. Graphical representation of elbow method to obtain optimal number of clusters for (A) clustering all insula areas in the 1000BRAINS cohort, (B) clustering all insula areas in the HCP data set, and (C) clustering the identified groups from approach (A). [file HBM-46-e70231-s005.pdf]

# A

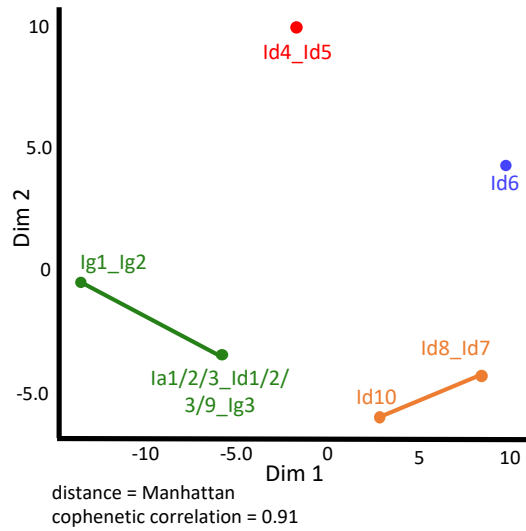

# B

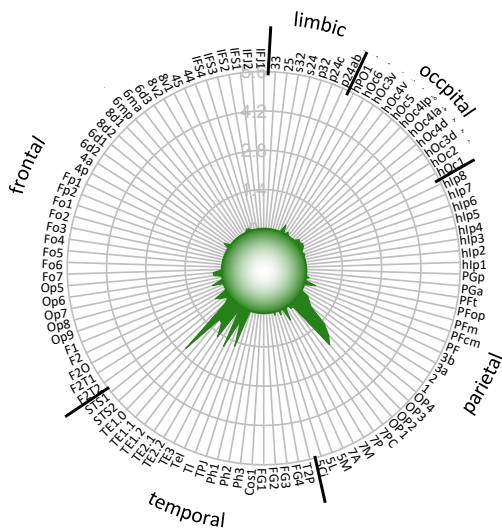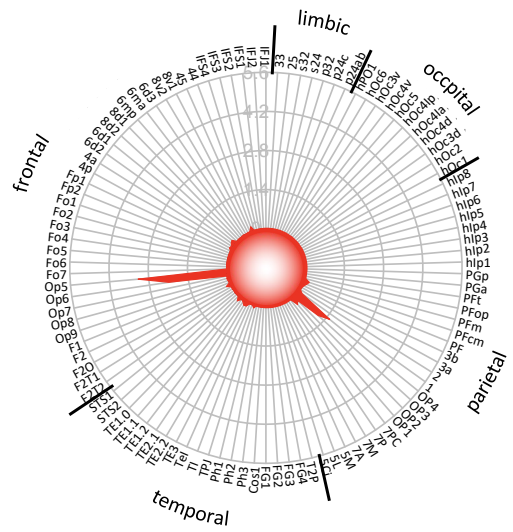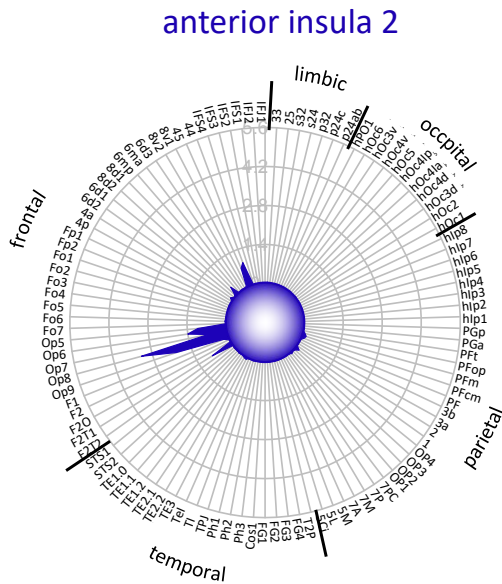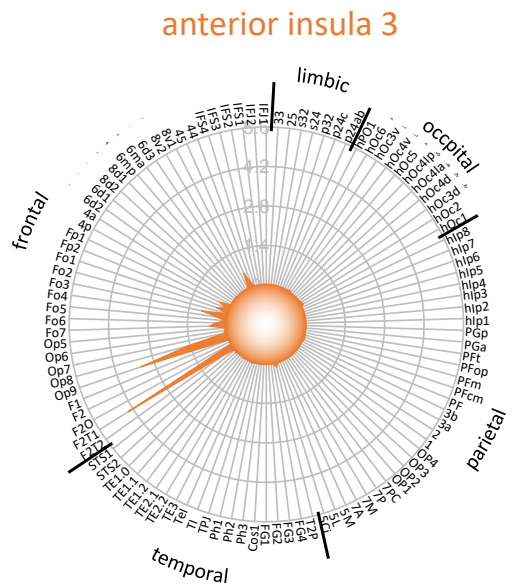

Supplement: Supplementary file 2 — Figure S2. Clustering of connectivity groups. Multidimensional scaling results in an optimal solution for k = 4 clusters (Figure S1). Posterior insula groups formed a cohesive cluster, whereas anterior insula groups exhibited less uniformity. Connectivity fingerprints for each cluster were depicted in (B). [file HBM-46-e70231-s007.pdf]

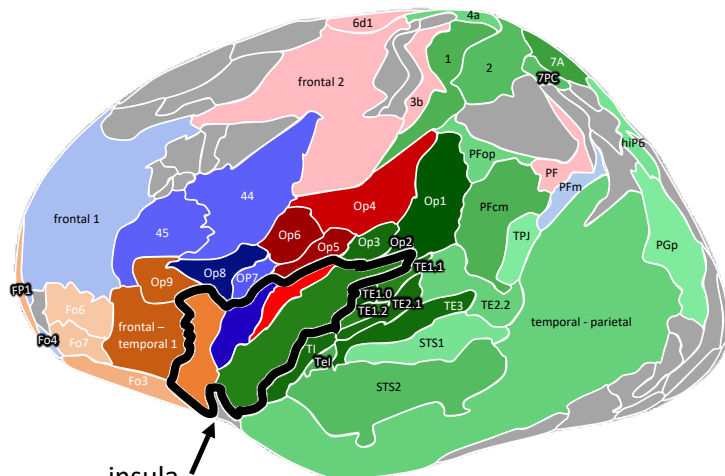

insula  
lateral view

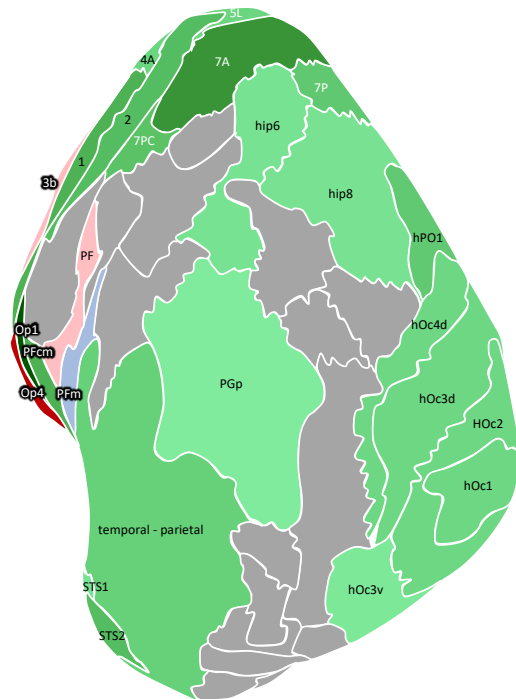

occipital view

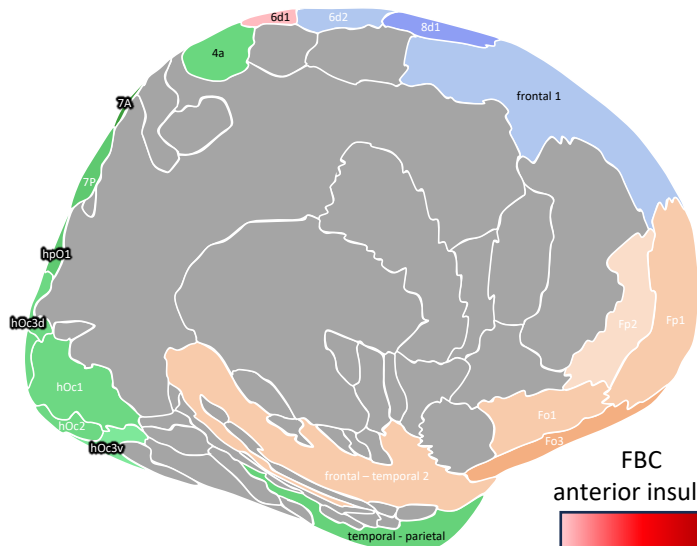

medial view

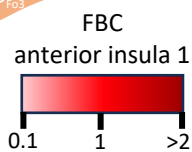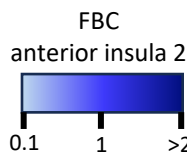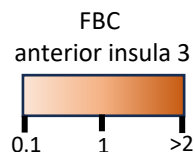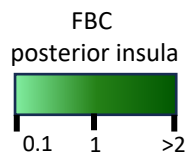

Supplement: Supplementary file 3 — Figure S3. Projection of clustered connectivity groups onto the Julich Brain fsaverage template. The color‐coded representation of areas signifies the predominant cluster with the highest fiber bundle capacity in the respective area. The intensity of coloring reflects the actual strength of connectivity. [file HBM-46-e70231-s003.pdf]

3D render

coronal

anterior posterior

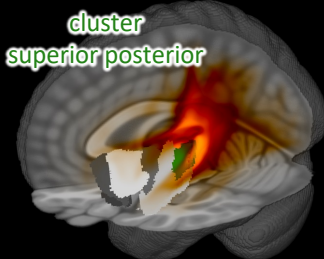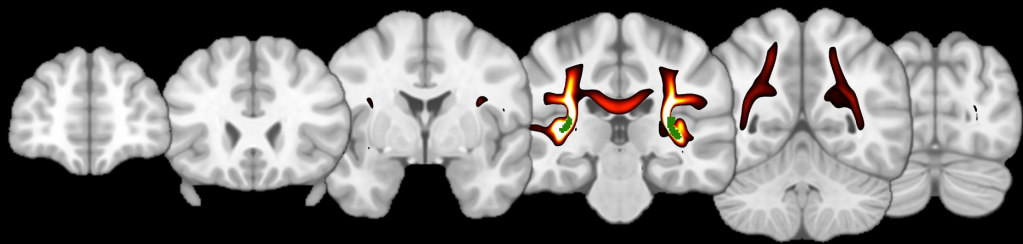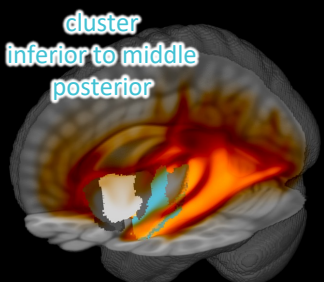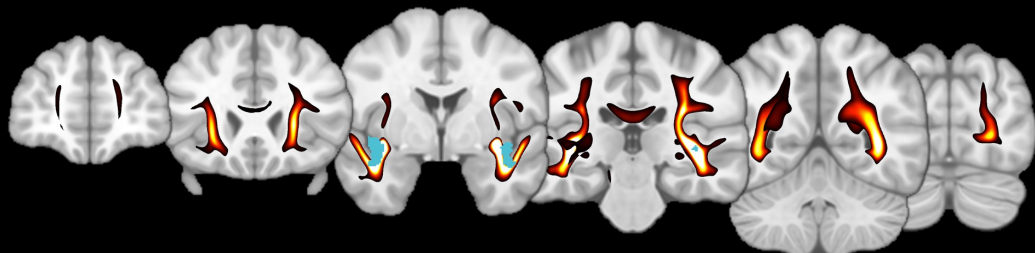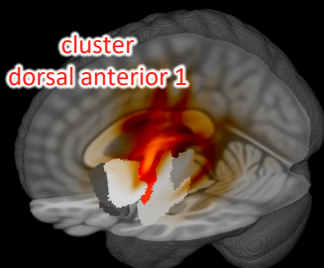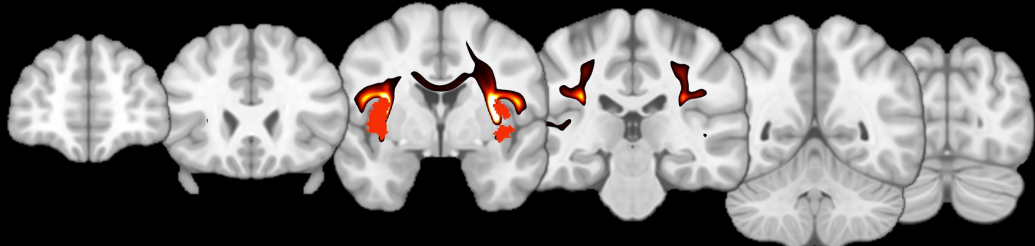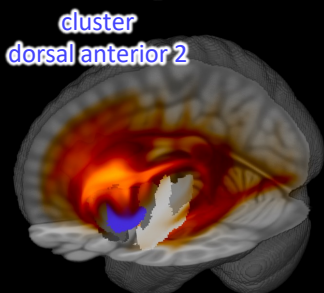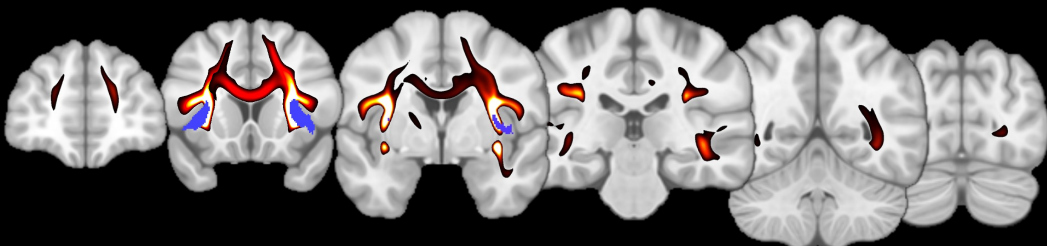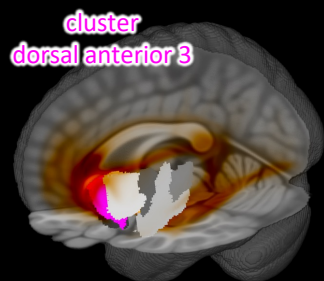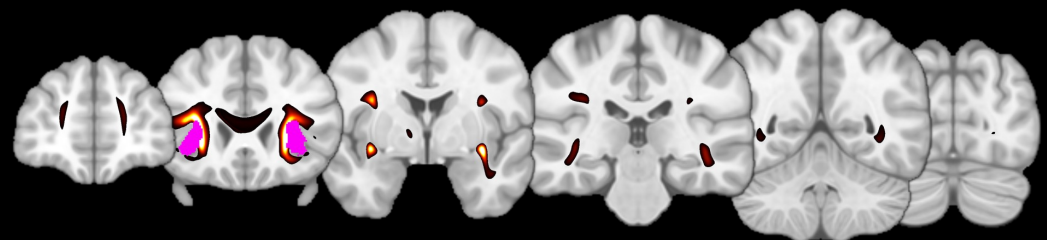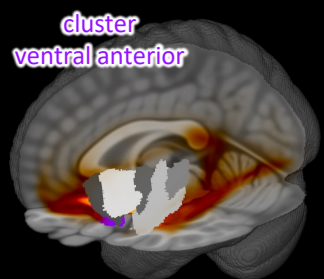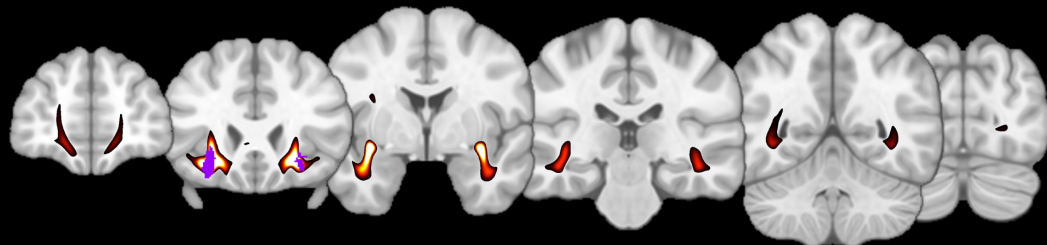

Supplement: Supplementary file 4 — Figure S4. Tract density maps for all insular connectivity clusters. Tract densities within each voxel are color‐coded, ranging from light red indicating high density to dark red representing lower density. [file HBM-46-e70231-s004.pdf]
