# Supplementary material for: Structural Connectivity Differences Reflect Microstructural Heterogeneity of the Human Insular Cortex
Source: Hum Brain Mapp. 2025 May 21;46(8):e70231. doi: 10.1002/hbm.70231 (PMC12093499; doi:10.1002/hbm.70231)
Supplement: Supplementary file 8 — Table S4. Conducting a t‐test, we examined the cluster with the highest connectivity strength for each anatomical and functional target region against the null hypothesis, positing that the connectivity strength does not differ from all other clusters. For each cluster combination, the analysis included reporting the corresponding effect size and the associated confidence interval. The cluster with the highest connectivity strength for each target area was considered significant (red caption) if the confidence interval of the effect size exceeded 0.8 for each cluster comparison. [file HBM-46-e70231-s009.docx]

|  | | **frontal – dorsal anterior 2** | **Broca – dorsal anterior 2** | **orbitofrontal – ventral anterior** | **motor – dorsal anterior 1** | **Auditory – superior posterior** |
| --- | --- | --- | --- | --- | --- | --- |
| dorsal anterior 1 | effect size d | 1.4 | 1.62 | 2.12 | - | 1.65 |
|  | 99% CI (d) | [1.21, 1.63] | [1.4, 1.83] | [1.88, 2.38] | - | [1.43, 1.87] |
| dorsal anterior 2 | effect size d | - | - | 2.1 | 0.64 | 1.74 |
|  | 99% CI (d) | - | - | [1.82, 2.31] | [0.46, 0.82] | [1.51, 1.96] |
| dorsal anterior 3 | effect size d | 0.34 | 0.49 | 1.76 | 1.23 | 1.76 |
|  | 99% CI (d) | [0.23, 0.57] | [0.31, 0.66] | [1.53, 1.99] | [1.02, 1.43] | [1.53, 1.99] |
| ventral anterior | effect size d | 2.5 | 1.86 | - | 1.26 | 1.76 |
|  | 99% CI (d) | [2.23, 2.77] | [1.63, 2.1] | - | [1.06, 1.47] | [1.54, 1.99] |
| superior posterior | effect size d | 3.6 | 1.83 | 2.13 | 0.77 | - |
|  | 99% CI (d) | [3.25, 3.96] | [1.61, 2.07] | [1.88, 2.37] | [0.6, 0.96] | - |
| inferior to middle posterior | effect size d | 2.61 | 1.68 | 1.61 | 0.96 | 1.4 |
|  | 99% CI (d) | [2.33, 2.89] | [1.46, 1.9] | [1.4, 1.83] | [0.77, 1.15] | [1.2, 1.61] |

**1000BRAINS**

|  | | **early visual – inferior to middle posterior** | **higher visual – inferior to middle posterior** | **prim. Somatosensory – superior posterior** | **seco. Somatosensory – superior posterior** |
| --- | --- | --- | --- | --- | --- |
| dorsal anterior 1 | effect size d | 1.65 | 1.86 | 0.59 | 0.48 |
|  | 99% CI (d) | [1.43, 1.87] | [1.62, 2.1] | [0.41, 0.76] | [0.30, 0.65] |
| dorsal anterior 2 | effect size d | 1.59 | 1.83 | 1.35 | 2.12 |
|  | 99% CI (d) | [1.37, 1.81] | [1.6, 2.1] | [1.14, 1.55] | [1.87, 2.37] |
| dorsal anterior 3 | effect size d | 1.51 | 1.75 | 1.42 | 2.33 |
|  | 99% CI (d) | [1.3, 1.73] | [1.52, 1.98] | [1.2, 1.6] | [2.07, 2.6] |
| ventral anterior | effect size d | 1.2 | 1.5 | 1.45 | 2.36 |
|  | 99% CI (d) | [1.0, 1.4] | [1.28, 1.70] | [1.24, 1.66] | [2.1, 2.6] |
| superior posterior | effect size d | 1.57 | 1.66 | - | - |
|  | 99% CI (d) | [1.35, 1.79] | [1.44, 1.88] | - | - |
| inferior to middle posterior | effect size d | - | - | 0.61 | 0.85 |
|  | 99% CI (d) | - | - | [0.43, 0.79] | [0.66, 1.03] |

**HCP**

|  | | **frontal – dorsal anterior 3** | **broca – dorsal anterior2** | **orbitofrontal – ventral anterior** | **motor – inferior to middel posterior** | **Auditory – superior posterior** |
| --- | --- | --- | --- | --- | --- | --- |
| dorsal anterior 1 | effect size d | 2.32 | 1.79 | 3.38 | 0.15 | 2.59 |
|  | 99% CI (d) | [1.77, 2.88] | [1.3, 2.28] | [2.66, 4.08] | [-0.21, 0.51] | [2.0, 3.18] |
| dorsal anterior 2 | effect size d | 0.68 | - | 3.25 | 0.12 | 2.6 |
|  | 99% CI (d) | [0.3, 1.06] | - | [2.57, 3.95] | [-0.24, 0.48] | [2.01, 3.2] |
| dorsal anterior 3 | effect size d | - | 0.15 | 2.58 | 1.65 | 2.62 |
|  | 99% CI (d) | - | [-0.2, 0.51] | [1.99, 3.17] | [1.18, 2.12] | [2.03, 3.22] |
| ventral anterior | effect size d | 1.03 | 2.63 | - | 1.72 | 2.61 |
|  | 99% CI (d) | [0.62, 1.43] | [2.07, 3.21] | - | [1.24, 2.19] | [2.02, 3.21] |
| superior posterior | effect size d | 3.71 | 3.22 | 3.35 | 1.34 | 0.24 |
|  | 99% CI (d) | [2.95, 4.48] | [2.54, 3.91] | [2.65, 4.05] | [0.9, 1.77] | [-0.12, 0.6] |
| inferior to middle posterior | effect size d | 1.21 | 2.58 | 2.8 | - | - |
|  | 99% CI (d) | [0.8, 1.64] | [1.99, 3.17] | [2.2, 3.42] | - | - |

|  | | **early visual – inferior to middle posterior** | **higher visual – inferior to middle posterior** | **prim. Somatosensory – inferior to middle posterior** | **seco. Somatosensory – superior posterior** |
| --- | --- | --- | --- | --- | --- |
| dorsal anterior 1 | effect size d | 2.34 | 2.71 | 0.19 | 0.9 |
|  | 99% CI (d) | [1.78, 2.9] | [2.12, 3.33] | [-0.16, 0.56] | [0.55, 1.34] |
| dorsal anterior 2 | effect size d | 2.29 | 2.73 | 1.23 | 2.98 |
|  | 99% CI (d) | [1.74, 2.85] | [2.12, 3.34] | [0.81, 1.66] | [2.34, 3.63] |
| dorsal anterior 3 | effect size d | 2.26 | 2.69 | 2.02 | 3.2 |
|  | 99% CI (d) | [1.72, 2.81] | [2.1, 3.3] | [1.51, 2.54] | [2.52, 3.88] |
| ventral anterior | effect size d | 1.87 | 2.48 | 2.07 | 3.18 |
|  | 99% CI (d) | [1.38, 2.37] | [1.9, 3.05] | [1.55, 2.58] | [2.5, 3.86] |
| superior posterior | effect size d | 2.18 | 2.34 | 1.15 | - |
|  | 99% CI (d) | [1.65, 2.72] | [1.78, 2.9] | [0.73, 1.57] | - |
| inferior to middle posterior | effect size d | - | - | - | 0.31 |
|  | 99% CI (d) | - | - | - | [-0.05, 0.68] |
